# Author Correction: Receptor-like cytoplasmic kinases of different subfamilies differentially regulate SOBIR1/BAK1-mediated immune responses in *Nicotiana benthamiana*

**DOI:** 10.1038/s41467-024-52322-5

**Published:** 2024-09-10

**Authors:** Wen R. H. Huang, Ciska Braam, Carola Kretschmer, Sergio Landeo Villanueva, Huan Liu, Filiz Ferik, Aranka M. van der Burgh, Sjef Boeren, Jinbin Wu, Lisha Zhang, Thorsten Nürnberger, Yulu Wang, Michael F. Seidl, Edouard Evangelisti, Johannes Stuttmann, Matthieu H. A. J. Joosten

**Affiliations:** 1 Laboratory of Phytopathology, Wageningen University, Droevendaalsesteeg 1, 6708 PB Wageningen, The Netherlands; 2 Institute for Biology, Department of Plant Genetics, Martin Luther University Halle-Wittenberg, 06120 Halle, Germany; 3 Laboratory of Biochemistry, Wageningen University and Research, Wageningen, the Netherlands; 4 Department of Plant Biochemistry, Centre for Plant Molecular Biology (ZMBP), Eberhard Karls University Tübingen, Auf der Morgenstelle 32, D-72076 Tübingen, Germany; 5 Laboratory of Biomanufacturing and Food Engineering, Institute of Food Science and Technology, Chinese Academy of Agricultural Sciences, Beijing, 100193 China; 6 Theoretical Biology & Bioinformatics, Department of Biology, Utrecht University, 3584 CH Utrecht, the Netherlands; 7 Aix Marseille University, CEA, CNRS, BIAM, UMR7265, LEMiRE (Microbial Ecology of the Rhizosphere), 13115 Saint‑Paul lez Durance, France; 8 Present Address: The Sainsbury Laboratory, University of East Anglia, Norwich, United Kingdom; 9 Present Address: Teaching and Learning Centre, Wageningen University & Research, Droevendaalsesteeg 4, 6708 PB Wageningen, The Netherlands; 10 Present Address: Department of Plant Pathology, Nanjing Agricultural University, Nanjing, 210095 China; 11 Present Address: Université Côte d’Azur, INRAE UMR 1355, CNRS UMR 7254, Institut Sophia Agrobiotech (ISA), 06903 Sophia Antipolis, France

**Keywords:** Plant molecular biology, Pattern recognition receptors in plants, Plant signalling, Kinases

Correction to: *Nature Communications* 10.1038/s41467-024-48313-1, published online 21 May 2024

The original version of this Article omitted from the author list the 8th author Sjef Boeren, who is from the Laboratory of Biochemistry, Wageningen University and Research, Wageningen, the Netherlands. Additionally, the following was added to the Author Contributions: ‘S.B. performed LC-MS/MS and assisted with the analysis of the acquired data.’

The original version of this Article contained an error in Results, which incorrectly read ‘*Sl*11220 in it turn us closely related to Arabidopsis CAS AWAY (*At*CST) (Supplementary Fig. 9), which is an RLCK involved in development that has indeed been shown to interact with SOBIR/EVR an negatively regulates signalling leading to cell separation^58^.’ The correct version states ‘*Sl*82500 and *Sl*112220 in their turn are closely related to Arabidopsis PBL30, PBL31 and PBL32, of which PBL30 (also known as CAST AWAY, CST) plays an important role in RLP1/SOBIR1-, RLP23/SOBIR1-, and RLP42/SOBIR1-mediated immunity (Supplementary Fig. 9)^36^. Both PBL30 and PBL31 have indeed been shown to interact with SOBIR1/EVR^36,58^.’ in place of ‘*Sl*11220 in it turn us closely related to Arabidopsis CAS AWAY (*At*CST) (Supplementary Fig. 9), which is an RLCK involved in development that has indeed been shown to interact with SOBIR/EVR an negatively regulates signalling leading to cell separation^58^’.

The original version of this Article contained an error in Results which incorrectly read ‘The Arabidopsis CERK1 is the co-receptor of the fungal cell wall component chitin^69,70^’ The correct version replaces this sentence with ‘Arabidopsis CERK1 is the co-receptor of the fungal cell wall component chitin^69,70^’.

The original version of this Article omitted four references to previous works.

‘Macho, A. P. et al. A bacterial tyrosine phosphatase inhibits plant pattern recognition receptor activation. Science 343, 1509–1512 (2014)’. This has been added as reference 96 at Discussion: For instance, upon the perception of elf18, the Arabidopsis LRR-RLK EFR phosphorylates at Tyr836, which is equivalent to NbSOBIR1 Tyr469, and this phosphorylation is required for the activation of EFR itself and the initiation of sequential downstream immune responses^96^’.

‘Macho, A. P. et al. A bacterial tyrosine phosphatase inhibits plant pattern recognition receptor activation. Science 343, 1509–1512 (2014); Perraki, A. et al. Phosphocode-dependent functional dichotomy of a common co-receptor in plant signalling. Nature 561, 248–252 (2018); Perraki, A. et al. Phosphocode-dependent functional dichotomy of a common co-receptor in plant signalling. Nature 561, 248–252 (2018); Mühlenbeck, H. et al. Allosteric activation of the co-receptor BAK1 by the EFR receptor kinase initiates immune signaling. eLife 12:RP92110 (2024)’. This has been added as reference 96-99 at Discussion: Strikingly, a vital role in regulating plant immunity has recently been assigned to this particular Tyr residue present in the kinase domain of several well-known RLKs^71,96-99’^.

The original version of this Article contained an error in the Methods section, which incorrectly omitted at the LC-MS/MS analysis section the following ‘For the LC–MS/MS analysis, the peptides were separated by reversed phase nano liquid chromatography, using a Thermo nLC1000 system equipped with a home-made C18 nanoLC column and they were measured using an Orbitrap Exploris 480 mass spectrometer.’

These have been corrected in the PDF and HTML versions of the Article.

