# Supplementary material for: Receptor-like cytoplasmic kinases of different subfamilies differentially regulate SOBIR1/BAK1-mediated immune responses in Nicotiana benthamiana
Source: Nat Commun. 2024 May 21;15:4339. doi: 10.1038/s41467-024-48313-1 (PMC11109355; doi:10.1038/s41467-024-48313-1)
Supplement: Supplementary file 1 — Supplementary Information [file 41467_2024_48313_MOESM1_ESM.pdf]

Supplemental information

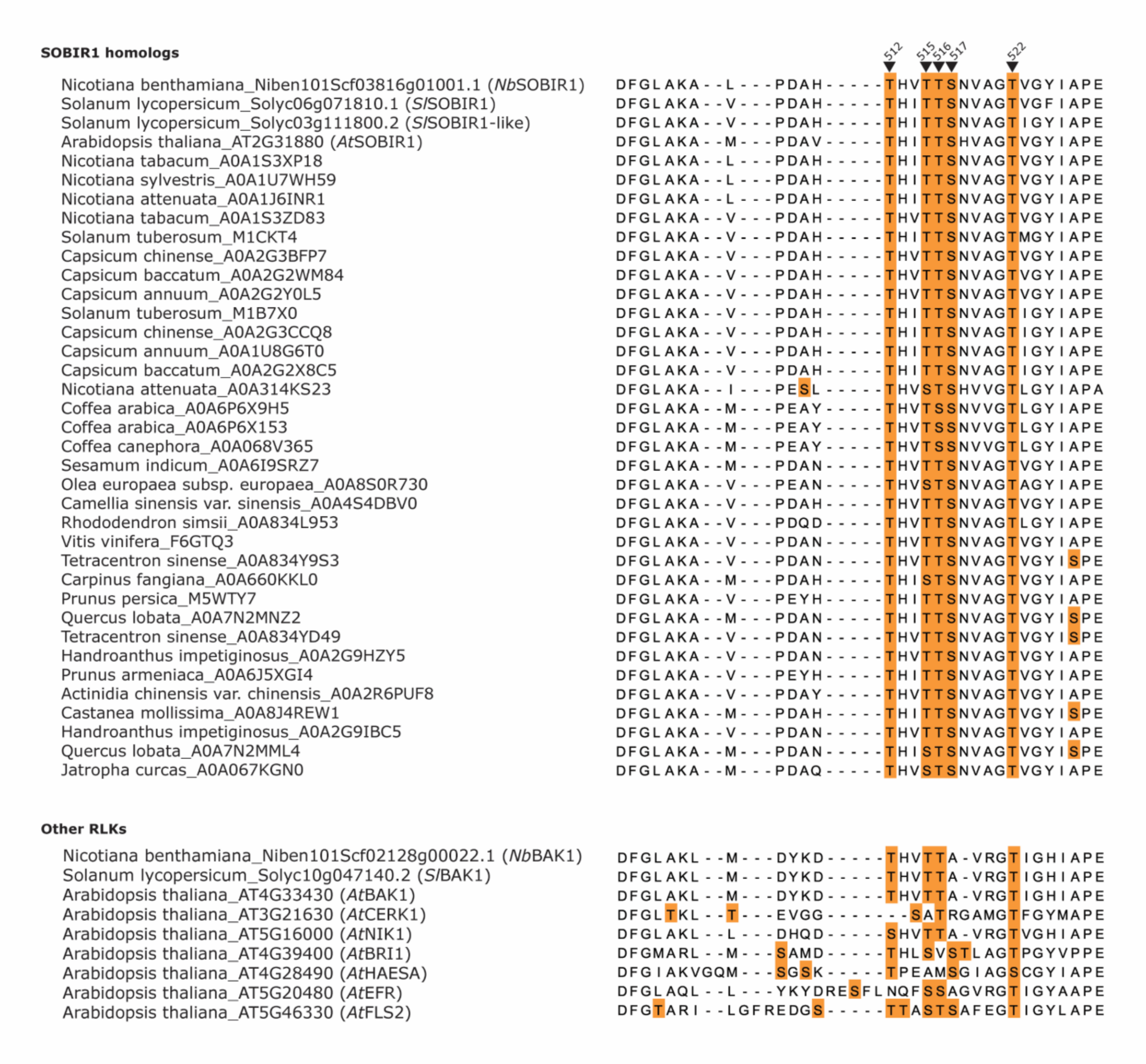

Supplementary Figure 1. Five potential phosphorylation sites in the activation segment of *Nb*SOBIR1 are highly conserved in the kinase domain of various RLKs from different plant species. Protein sequences of the SOBIR1 homologs from various plant species were obtained from the UniProt database (https://www.uniprot.org/), while the protein sequences of other RLKs were retrieved from TAIR (https://www.arabidopsis.org/index.jsp) and the Sol Genomics Network (https://solgenomics.net/). The alignment was visualized using JalView and only the amino acid sequences of the activation segment of the kinase domain are shown. All the putatively phosphorylatable Ser (S) and Thr (T) residues are highlighted in orange. The positions of the five Ser/Thr residues of *Nb*SOBIR1 that are subjected to this study are indicated on top.

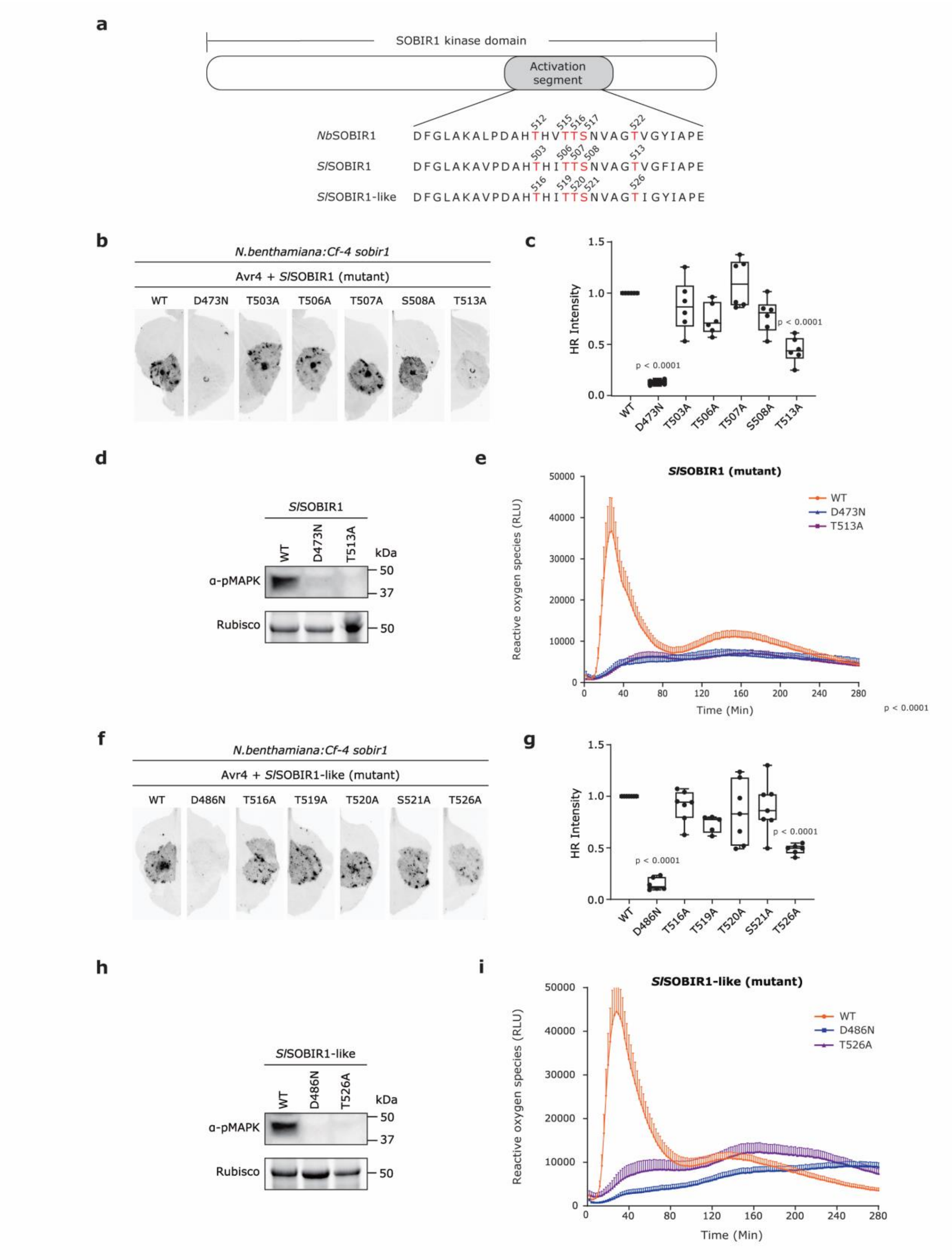

Supplementary Figure 2. The analogous residues of *Nb*SOBIR1 Thr522 in both tomato SOBIR1 and SOBIR1-like play a crucial role in mounting Avr4/Cf-4-triggered immune responses. a, Schematic diagram of the kinase domain of SOBIR1, with the activation segment indicated. The amino acid sequences of the activation segments of *Nb*SOBIR1, *Sl*SOBIR1 and

*Sl*SOBIR1-like are aligned and are shown below the diagram. Conserved residues acting as potential phosphorylation sites are indicated in red. **b**-**e**, Mutagenesis screen of five potential phosphorylation sites in the activation segment of *Sl*SOBIR1 based on the Avr4/Cf-4-triggered HR activation (**b** and **c**), MAPK activation (**d**), and ROS burst (**e**). **f**-**i**, Mutagenesis screen of five potential phosphorylation sites in the activation segment of *Sl*SOBIR1-like based on the Avr4/Cf-4-triggered HR activation (**f** and **g**), MAPK activation (**h**), and ROS burst (**i**). The development of an HR was imaged at 5 dpi by the Chemidoc (**b** and **f**), and the HR intensity was quantified by Image Lab (**c** and **g**). The HR intensity of leaves that transiently co-express *SlSOBIR1 WT* with *Avr4* (**c**) or *SlSOBIR1-like WT* with *Avr4* (**g**) was set as 1. Statistical analysis was performed with an ANOVA/Dunnett's multiple comparison test, compared with their corresponding WT. Dots indicate individual values (centre line, median; error bar, minima and maxima; n=6). **e**, **i**, Leaf discs of *N. benthamiana:Cf-4 sobir1* knock-out plants that transiently express individual *Sl*SOBIR1 and *Sl*SOBIR1-like mutants, as well as their corresponding WTs and kinase-dead mutants, were treated with 0.1 μM Avr4 protein, followed by measuring the production of ROS. ROS production is expressed as RLUs, and the data are represented as mean + SEM (n=12). Similar to SOBIR1 WT, all tested SOBIR1 Ser/Thr mutants restored the Avr4/Cf-4-triggered ROS production in this complementation study, except for *Sl*SOBIR1 T513A and *Sl*SOBIR1-like T526A. Only the results for *Sl*SOBIR1 WT, T513A and D473N are shown in (**e**), and the results for *Sl*SOBIR1-like WT, T526A, and D486N are shown in (**i**). Experiments were repeated at least three times, with similar results. Source data are provided as a Source Data file.

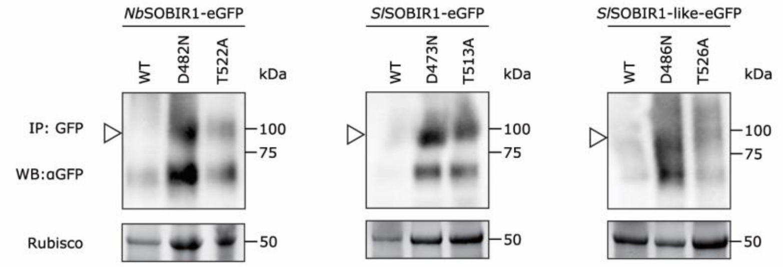

Supplementary Figure 3. Accumulation levels of *Nb*SOBIR1 T522A, *Sl*SOBIR1 T513A and *Sl*SOBIR1-like T526A *in planta*, in comparison to their WT and kinase-dead ("RN") versions. *NbSOBIR1 T522A*, *SlSOBIR1 T513A* and *SlSOBIR1-like T526A*, which fail to restore the Avr4-triggered HR in *N. benthamiana:Cf-4 sobir1* knock-out plants, were transiently co-expressed in *N. benthamiana:Cf-4 sobir1* plants with *Avr4* (both at an OD_{600} of 0.8). Transient expression of their respective WT and kinase-dead D to N version, in combination with *Avr4*, was included as positive and negative controls, respectively. Leaf samples were collected at 2 dpi and total protein extracts were subjected to immuno-purification (IP) using GFP-affinity beads, followed by western blotting (WB) with αGFP antibody (upper panels). The amount of total protein that was used for the IP is reflected by the Rubisco band present in the total protein extracts loaded on SDS gel (lower panels). Arrowheads indicate the band corresponding to SOBIR1-eGFP. Note that transient expression of *WT SOBIR1* in combination with *Avr4* results in cell death, thereby causing very low WT SOBIR1 accumulation levels.

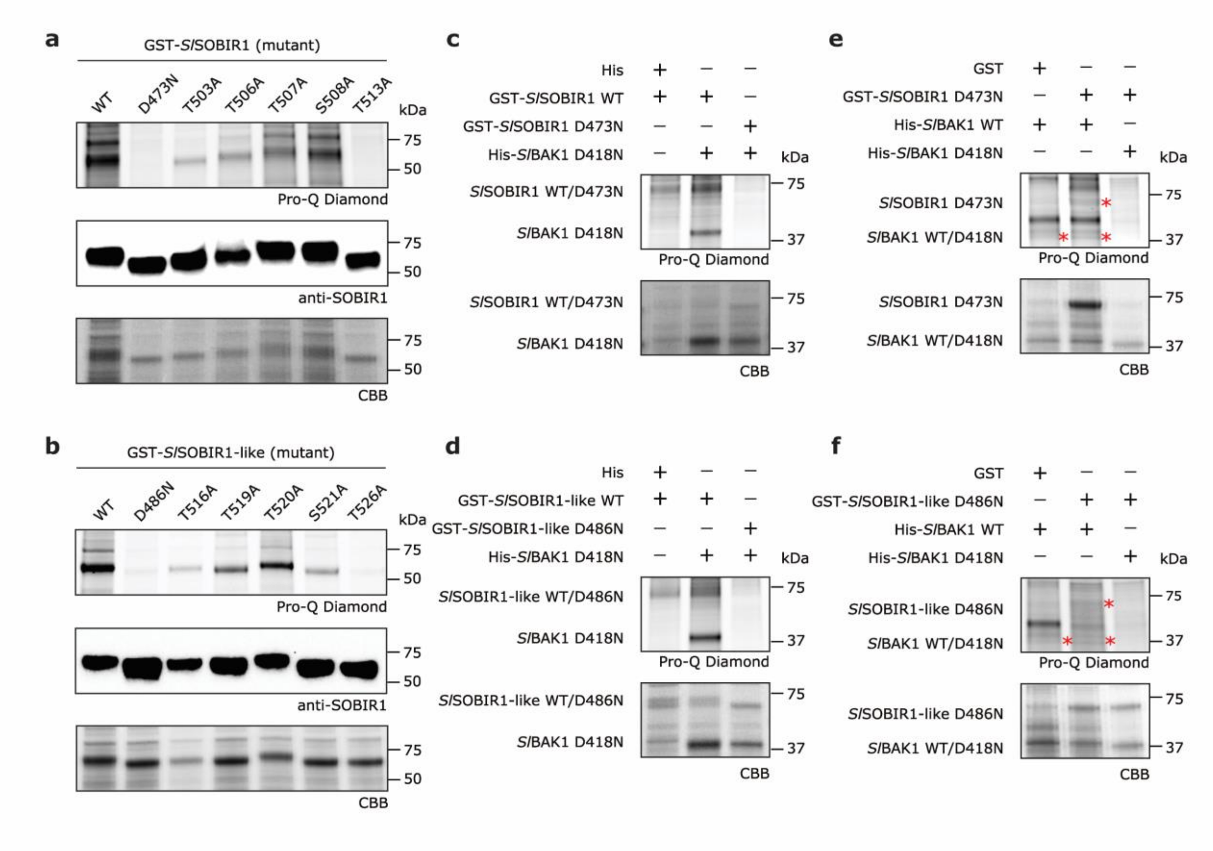

Supplementary Figure 4. The kinase domains of SOBIR1 and BAK1 trans-phosphorylate each other *in vitro*. a, **b**, *Sl*SOBIR1 Thr513 and *Sl*SOBIR1-like Thr526 are essential for their intrinsic kinase activity. The N-terminally GST-tagged cytoplasmic kinase domains of (**a**) *Sl*SOBIR1 WT, kinase-dead mutant D473N, and five Ser/Thr-to Ala mutants, and (**b**) *Sl*SOBIR1-like WT, kinase-dead mutant D486N, and five Ser/Thr-to-Ala mutants were produced in *E. coli*, followed by being subjected to western blotting and *in vitro* phosphorylation assay. The phosphorylation status of the recombinant proteins was determined by using the Pro-Q Diamond stain, which specifically stains the phosphorylated proteins (top panels). The production of SOBIR1 kinase domains was confirmed by western blotting, using SOBIR1 antibodies (middle panels), and the recombinant proteins were stained by Coomassie brilliant blue (bottom panels). **c**,**d**, *Sl*SOBIR1 WT and *Sl*SOBIR1-like WT directly phosphorylate the kinase-dead mutant of *Sl*BAK1 D418N. The N-terminally GST-tagged cytoplasmic domains of *Sl*SOBIR1 WT or D473N (**c**) and *Sl*SOBIR1-like WT or D486N (**d**) were co-expressed with the N-terminally His-tagged cytoplasmic domain of *Sl*BAK1 D418N in *E. coli*. After SDS-PAGE of the *E. coli* lysates, a Pro-Q Diamond stain was employed to detect the phosphorylated recombinant proteins (top panels), while Coomassie brilliant blue was used to visualize all proteins (bottom panels). **e**,**f**, *Sl*BAK1 WT directly phosphorylates the kinase-dead mutant of *Sl*SOBIR1 D473N and *Sl*SOBIR1-like D486N. The cytoplasmic domain of *Sl*BAK1 WT or D418N, which was fused to a His tag at its N-terminus, was co-expressed with the cytoplasmic domain of *Sl*SOBIR1 D473N (**e**) or *Sl*SOBIR1-like D486N (**f**), which was fused to a GST tag at its N-terminus, in *E. coli*. The recombinant proteins were then subjected to SDS-PAGE, followed by being stained by Pro-Q Diamond stain (top panels) and Coomassie brilliant blue (bottom panels). Bands with the expected sizes are indicated with an asterisk.

Experiments were repeated at least three times with similar results, and representative pictures are shown. Source data are provided as a Source Data file.

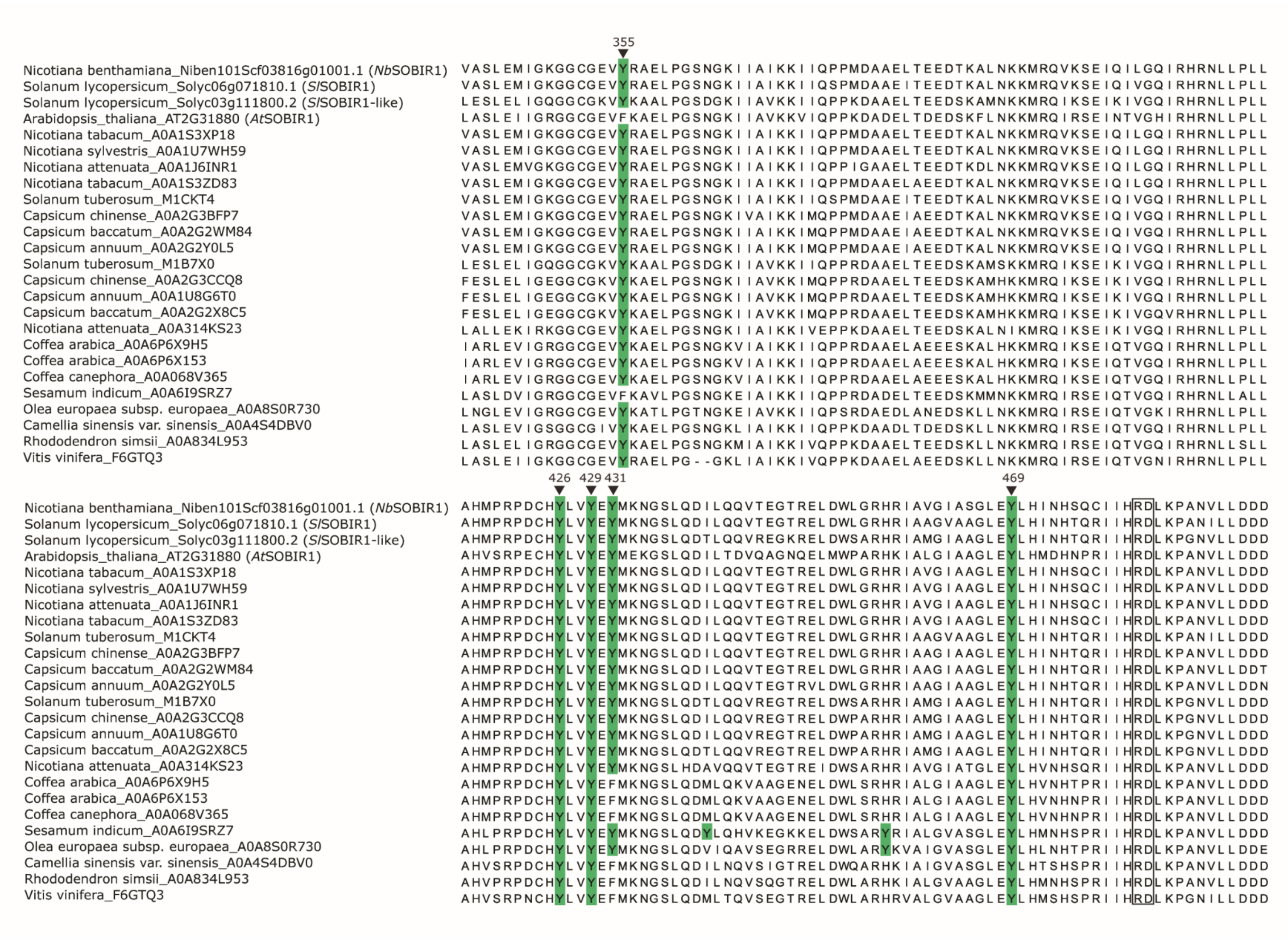
355
Nicotiana benthamiana_Niben101Scf03816g01001.1 (NbSOBIR1) VASLEMIGKGGCGEVYRAELPGSNGKIIAIKKIIQPPMDAAELTEEDTKALNKKMRQVKSEIQILGQIRHRNLLPLL
Solanum lycopersicum_Solyc06g071810.1 (SlSOBIR1) VASLEMIGKGGCGEVYRAELPGSNGKIIAIKKIIQSPMDAAEITEEDTKALNKKMRQVKSEIQIVGQIRHRNLLPLL
Solanum lycopersicum_Solyc03g111800.2 (SlSOBIR1-like) LESLELIGQGGCGKVYKAALPGSDGKIIAVKKIIQPPKDAAELTEEDSKAMNKKMRQIKSEIKIVGQIRHRNLLPLL
Arabidopsis_thaliana_AT2G31880 (AtSOBIR1) LASLEIIGRGGCGEVFKAELPGSNGKIIAVKKVIQPPKDADELTDEDSKFLNKKMRQIRSEINTVGHIRHRNLLPLL
Nicotiana tabacum_A0A1S3XP18 VASLEMIGKGGCGEVYRAELPGSNGKIIAIKKIIQPPMDAAELTEEDTKALNKKMRQVKSEIQILGQIRHRNLLPLL
Nicotiana sylvestris_A0A1U7WH59 VASLEMIGKGGCGEVYRAELPGSNGKIIAIKKIIQPPMDAAELTEEDTKALNKKMRQVKSEIQILGQIRHRNLLPLL
Nicotiana attenuata_A0A1J6INR1 VASLEMVGKGGCGEVYRAELPGSNGKIIAIKKIIQPPIGAAELTEEDTKDLNKKMRQVKSEIQILGQIRHRNLLPLL
Nicotiana tabacum_A0A1S3ZD83 VASLEMIGKGGCGEVYRAELPGSNGKIIAIKKIIQPPMDAAELAEEDTKALNKKMRQVKSEIQILGQIRHRNLLPLL
Solanum tuberosum_M1CKT4 VASLEMIGKGGCGEVYRAELPGSNGKIIAIKKIIQSPMDAAEITEEDTKALNKKMRQVKSEIQIVGQIRHRNLLPLL
Capsicum chinense_A0A2G3BFP7 VASLEMIGKGGCGEVYRAELPGSNGKIVAIKKIMQPPMDAAEIAEEDTKALNKKMRQVKSEIQIVGQIRHRNLLPLL
Capsicum baccatum_A0A2G2WM84 VASLEMIGKGGCGEVYRAELPGSNGKIIAIKKIMQPPMDAAEIAEEDTKALNKKMRQVKSEIQIVGQIRHRNLLPLL
Capsicum annuum_A0A2G2Y0L5 VASLEMIGKGGCGEVYRAELPGSNGKIIAIKKIMQPPMDAAEIAEEDTKALNKKMRQVKSEIQIVGQIRHRNLLPLL
Solanum tuberosum_M1B7X0 LESLELIGQGGCGKVYKAALPGSDGKIIAVKKIIQPPRDAAELTEEDSKAMSKKMRQIKSEIKIVGQIRHRNLLPLL
Capsicum chinense_A0A2G3CCQ8 FESLELIGEGGCGKVYKAELPGSNGKIIAVKKIMQPPRDAAELTEEDSKAMHKKMRQIKSEIKIVGQIRHRNLLPLL
Capsicum annuum_A0A1U8G6T0 FESLELIGEGGCGKVYKAELPGSNGKIIAVKKIMQPPRDAAELTEEDSKAMHKKMRQIKSEIKIVGQIRHRNLLPLL
Capsicum baccatum_A0A2G2X8C5 FESLELIGEGGCGKVYKAELPGSNGKIIAVKKIMQPPRDAAELTEEDSKAMHKKMRQIKSEIKIVGQVRHRNLLPLL
Nicotiana attenuata_A0A314KS23 LALLEKIRKGGCGEVYKAELPGSNGKIIAIKKIVEPPKDAAELTEEDSKALNIKMRQIKSEIKIVGQIRHRNLLPLL
Coffea arabica_A0A6P6X9H5 IARLEVIGRGGCGEVYKAELPGSNGKVIAIKKIIQPPRDAAELAEEESKALHKKMRQIKSEIQTVGQIRHRNLLPLL
Coffea arabica_A0A6P6X153 IARLEVIGRGGCGEVYKAELPGSNGKVIAIKKIIQPPRDAAELAEEESKALHKKMRQIKSEIQTVGQIRHRNLLPLL
Coffea canephora_A0A068V365 IARLEVIGRGGCGEVYKAELPGSNGKVIAIKKIIQPPRDAAELAEEESKALHKKMRQIKSEIQTVGQIRHRNLLPLL
Sesamum indicum_A0A6I9SRZ7 LASLDVIGRGGCGEVFKAVLPGSNGKEIAIKKIIQPPRDADELTEEDSKMMNKKMRQIRSEIQTVGQIRHRNLLALL
Olea europaea subsp. europaea_A0A8S0R730 LNGLEVIGRGGCGEVYKATLPGTNGKEIAVKKIIQPSRDAEDLANEDSKLLNKKMRQIRSEIQTVGKIRHRNLLPLL
Camellia sinensis var. sinensis_A0A4S4DBV0 LASLEVIGSGGCGIVYKAELPGSNGKLIAIKKIIQPPKDAADLTDEDSKLLNKKMRQIRSEIQTVGQIRHRNLLPLL
Rhododendron simsii_A0A834L953 LASLELIGRGGCGEVYKAELPGSNGKMIAIKKIVQPPKDAAELTEEDSKLLNKKMRQIRSEIQTVGQIRHRNLLSLL
Vitis vinifera_F6GTQ3 LASLEIIGKGGCGEVYRAELPG--GKLIAIKKIVQPPKDAAELAEEDSKLLNKKMRQIRSEIQTVGNIRHRNLLPLL
426 429 431 469
Nicotiana benthamiana_Niben101Scf03816g01001.1 (NbSOBIR1) AHMPRPDCHYLVYEYMKNGSLQDILQQVTEGTRELDWLGRHRIAVGIASGLEYLHINHSQCIIHRDLKPANVLLDDD
Solanum lycopersicum_Solyc06g071810.1 (SlSOBIR1) AHMPRPDCHYLVYEYMKNGSLQDILQQVTEGTRELDWLGRHRIAAGVAAGLEYLHINHTQRIIHRDLKPANILLDDD
Solanum lycopersicum_Solyc03g111800.2 (SlSOBIR1-like) AHMPRPDCHYLVYEYMKNGSLQDTLQQVREGKRELDWSARHRIAMGIAAGLEYLHINHTQRIIHRDLKPGNVLLDDD
Arabidopsis_thaliana_AT2G31880 (AtSOBIR1) AHVSRPECHYLVYEYMEKGSLQDILTDVQAGNQELMWPARHKIALGIAAGLEYLHMDHNPRIIHRDLKPANVLLDDD
Nicotiana tabacum_A0A1S3XP18 AHMPRPDCHYLVYEYMKNGSLQDILQQVTEGTRELDWLGRHRIAVGIAAGLEYLHINHSQCIIHRDLKPANVLLDDD
Nicotiana sylvestris_A0A1U7WH59 AHMPRPDCHYLVYEYMKNGSLQDILQQVTEGTRELDWLGRHRIAVGIAAGLEYLHINHSQCIIHRDLKPANVLLDDD
Nicotiana attenuata_A0A1J6INR1 AHMPRPDCHYLVYEYMKNGSLQDILQQVTEGTRELDWLGRHRIAVGIAAGLEYLHINHSQCIIHRDLKPANVLLDDD
Nicotiana tabacum_A0A1S3ZD83 AHMPRPDCHYLVYEYMKNGSLQDILQQVTEGTRELDWLGRHRIAVGIAAGLEYLHINHSQCIIHRDLKPANVLLDDD
Solanum tuberosum_M1CKT4 AHMPRPDCHYLVYEYMKNGSLQDILQQVTEGTRELDWLGRHRIAAGVAAGLEYLHINHTQRIIHRDLKPANILLDDD
Capsicum chinense_A0A2G3BFP7 AHMPRPDCHYLVYEYMKNGSLQDILQQVTEGTRELDWLGRHRIAAGIAAGLEYLHINHTQRIIHRDLKPANVLLDDD
Capsicum baccatum_A0A2G2WM84 AHMPRPDCHYLVYEYMKNGSLQDILQQVTEGTRELDWLGRHRIAAGIAAGLEYLHINHTQRIIHRDLKPANVLLDDT
Capsicum annuum_A0A2G2Y0L5 AHMPRPDCHYLVYEYMKNGSLQDILQQVTEGTRVLDWLGRHRIAAGIAAGLEYLHINHTQRIIHRDLKPANVLLDDN
Solanum tuberosum_M1B7X0 AHMPRPDCHYLVYEYMKNGSLQDTLQQVREGTRELDWSARHRIAMGIAAGLEYLHINHTQRIIHRDLKPGNVLLDDD
Capsicum chinense_A0A2G3CCQ8 AHMPRPDCHYLVYEYMKNGSLQDILQQVREGTRELDWPARHRIAMGIAAGLEYLHINHTQRIIHRDLKPGNVLLDDD
Capsicum annuum_A0A1U8G6T0 AHMPRPDCHYLVYEYMKNGSLQDILQQVREGTRELDWPARHRIAMGIAAGLEYLHINHTQRIIHRDLKPGNVLLDDD
Capsicum baccatum_A0A2G2X8C5 AHMPRPDCHYLVYEYMKNGSLQDTLQQVREGTRELDWPARHRIAMGIAAGLEYLHINHTQRIIHRDLKPGNVLLDDD
Nicotiana attenuata_A0A314KS23 AHMPRPDCHYLVYEYMKNGSLHDAVQQVTEGTREIDWSARHRIAVGIATGLEYLHVNHSQRIIHRDLKPANVLLDDD
Coffea arabica_A0A6P6X9H5 AHMPRPDCHYLVYEFMKNGSLQDMLQKVAAGENELDWLSRHRIALGIAAGLEYLHVNHTPRIIHRDLKPANVLLDDD
Coffea arabica_A0A6P6X153 AHMPRPDCHYLVYEFMKNGSLQDMLQKVAAGENELDWLSRHRIALGIAAGLEYLHVNHNPRIIHRDLKPANVLLDDD
Coffea canephora_A0A068V365 AHMPRPDCHYLVYEFMKNGSLQDMLQKVAAGENELDWLSRHRIALGIAAGLEYLHVNHNPRIIHRDLKPANVLLDDD
Sesamum indicum_A0A6I9SRZ7 AHLPRPDCHYLVYEYMKNGSLQDYLQHVKEGKKELDWSARYRIALGVASGLEYLHMNHSPRIIHRDLKPANVLLDDD
Olea europaea subsp. europaea_A0A8S0R730 AHLPRPDCHYLVYEYMKNGSLQDVIQAVSEGRRELDWLARYKVAIGVASGLEYLHLNHTPRIIHRDLKPANVLLDDE
Camellia sinensis var. sinensis_A0A4S4DBV0 AHVSRPDCHYLVYEFMKNGSLQDILNQVSIGTRELDWQARHKIAIGVASGLEYLHTSHSPRIIHRDLKPANVLLDDD
Rhododendron simsii_A0A834L953 AHVPRPDCHYLVYEFMKNGSLQDILNQVSQGTRELDWLARHKIALGVAAGLEYLHMNHSPRIIHRDLKPANVLLDDD
Vitis vinifera_F6GTQ3 AHVSRPNCHYLVYEFMKNGSLQDMLTQVSEGTRELDWLARHRVALGVAAGLEYLHMSHSPRIIHRDLKPGNILLDDD

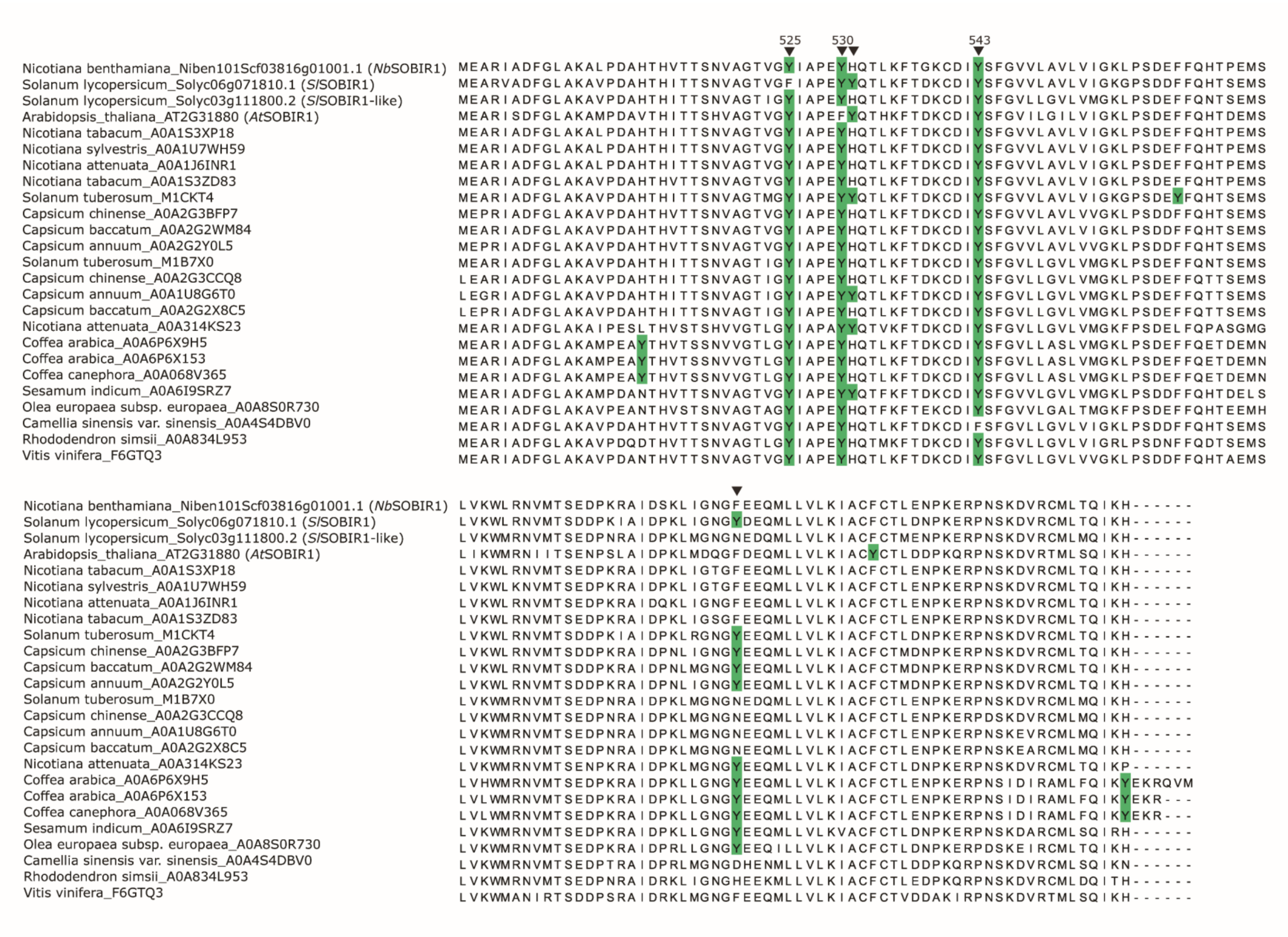
525
530
543
Nicotiana benthamiana_Niben101Scf03816g01001.1 (NbSOBIR1) MEARIADFGLAKALPDAHTHVTTSNVAGTVGYIAPEYHQTLKFTGKCDIYSFGVVLAVLVIGKLPSDEFFQHTPEMS
Solanum lycopersicum_Solyc06g071810.1 (SlSOBIR1) MEARVADFGLAKAVPDAHTHITTSNVAGTVGFIAPEYYQTLKFTDKCDIYSFGVVLAVLVIGKGPSDDFFQHTSEMS
Solanum lycopersicum_Solyc03g111800.2 (SlSOBIR1-like) MEARIADFGLAKAVPDAHTHITTSNVAGTIGYIAPEYHQTLKFTDKCDIYSFGVVLLGVLVMGKLPSDEFFQNTSEMS
Arabidopsis_thaliana_AT2G31880 (AtSOBIR1) MEARISDFGLAKAMPDAVTHITTSHVAGTVGYIAPEFYQTHKFTDKCDIYSFGVILGILVIGKLPSDEFFQHTDEMS
Nicotiana tabacum_A0A1S3XP18 MEARIADFGLAKALPDAHTHITTSNVAGTVGYIAPEYHQTLKFTDKCDIYSFGVVLAVLVIGKLPSDEFFQHTPEMS
Nicotiana sylvestris_A0A1U7WH59 MEARIADFGLAKALPDAHTHITTSNVAGTVGYIAPEYHQTLKFTDKCDIYSFGVVLAVLVIGKLPSDEFFQHTPEMS
Nicotiana attenuata_A0A1J6INR1 MEARIADFGLAKALPDAHTHITTSNVAGTVGYIAPEYHQTLKFTDKCDIYSFGVVLAVLVIGKLPSDEFFQHTPEMS
Nicotiana tabacum_A0A1S3ZD83 MEARIADFGLAKAVPDAHTHVTTSNVAGTVGYIAPEYHQTLKFTDKCDIYSFGVVLAVLVIGKLPSDEFFQHTPEMS
Solanum tuberosum_M1CKT4 MEARIADFGLAKAVPDAHTHITTSNVAGTMGYIAPEYYQTLKFTDKCDIYSFGVVLAVLVIGKGPSDEYFQHTSEMS
Capsicum chinense_A0A2G3BFP7 MEPRIADFGLAKAVPDAHTHVTTSNVAGTVGYIAPEYHQTLKFTDKCDIYSFGVVLAVLVVGKLPSDDFFQHTSEMS
Capsicum baccatum_A0A2G2WM84 MEARIADFGLAKAVPDAHTHVTTSNVAGTVGYIAPEYHQTLKFTDKCDIYSFGVVLAVLVIGKLPSDDFFQHTSEMS
Capsicum annuum_A0A2G2Y0L5 MEPRIADFGLAKAVPDAHTHVTTSNVAGTVGYIAPEYHQTLKFTDKCDIYSFGVVLAVLVVGKLPSDDFFQHTSEMS
Solanum tuberosum_M1B7X0 MEARIADFGLAKAVPDAHTHITTSNVAGTIGYIAPEYHQTLKFTDKCDIYSFGVLLGVLVMGKLPSDEFFQNTSEMS
Capsicum chinense_A0A2G3CCQ8 LEARIADFGLAKAVPDAHTHITTSNVAGTIGYIAPEYHQTLKFTDKCDIYSFGVLLGVLVMGKLPSDEFFQTTSEMS
Capsicum annuum_A0A1U8G6T0 LEGRIADFGLAKAVPDAHTHITTSNVAGTIGYIAPEYYQTLKFTDKCDIYSFGVLLGVLVMGKLPSDEFFQTTSEMS
Capsicum baccatum_A0A2G2X8C5 LEPRIADFGLAKAVPDAHTHITTSNVAGTIGYIAPEYHQTLKFTDKCDIYSFGVLLGVLVMGKLPSDEFFQTTSEMS
Nicotiana attenuata_A0A314KS23 MEARIADFGLAKAIPESLTHVSTSHVVGTLGYIAPAYYQTVKFTDKCDIYSFGVLLGVLVMGKFPSDELFQPASGMG
Coffea arabica_A0A6P6X9H5 MEARIADFGLAKAMPEAYTHVTSSNVVGTLGYIAPEYHQTLKFTDKCDIYSFGVLLASLVMGKLPSDEFFQETDEMN
Coffea arabica_A0A6P6X153 MEARIADFGLAKAMPEAYTHVTSSNVVGTLGYIAPEYHQTLKFTDKCDIYSFGVLLASLVMGKLPSDEFFQETDEMN
Coffea canephora_A0A068V365 MEARIADFGLAKAMPEAYTHVTSSNVVGTLGYIAPEYHQTLKFTDKCDIYSFGVLLASLVMGKLPSDEFFQETDEMN
Sesamum indicum_A0A6I9SRZ7 MEARIADFGLAKAMPDANTHVTTSNVAGTVGYIAPEYYQTFKFTDKCDIYSFGVVLGVLVMGKLPSDDFFQHTDELS
Olea europaea subsp. europaea_A0A8S0R730 MEARIADFGLAKAVPEANTHVSTSNVAGTAGYIAPEYHQTFKFTEKCDIYSFGVVLGALTMGKFPSDEFFQHTEEMH
Camellia sinensis var. sinensis_A0A4S4DBV0 MEARIADFGLAKAVPDAHTHVTTSNVAGTVGYIAPEYHQTLKFTDKCDIFSFGVVLAVLVIGKLPSDDFFQHTSEMS
Rhododendron simsii_A0A834L953 MEARIADFGLAKAVPDQDTHVTTSNVAGTLGYIAPEYHQTMKFTDKCDIYSFGVLLGVLVIGRLPSDNFFQDTSEMS
Vitis vinifera_F6GTQ3 MEARIADFGLAKAVPDANTHVTTSNVAGTVGYIAPEYHQTLKFTDKCDIYSFGVLLGVLVVGKLPSDDFFQHTAEMS
Nicotiana benthamiana_Niben101Scf03816g01001.1 (NbSOBIR1) LVKWLRNVMTSEDPKRAIDSKLIGNGFEEQMLLVLKIACFCTLENPKERPNSKDVRCMLTQIKH------
Solanum lycopersicum_Solyc06g071810.1 (SlSOBIR1) LVKWLRNVMTSDDPKIAIDPKLIGNGYDEQMLLVLKIACFCTLDNPKERPNSKDVRCMLTQIKH------
Solanum lycopersicum_Solyc03g111800.2 (SlSOBIR1-like) LVKWMRNVMTSEDPNRAIDPKLMGNGNEDQMLLVLKIACFCTMENPKERPNSKDVRCMLMQIKH------
Arabidopsis_thaliana_AT2G31880 (AtSOBIR1) LIKWMRNIITSENPSLAIDPKLMDQGFDEQMLLVLKIACYCTLDDPKQRPNSKDVRTMLSQIKH------
Nicotiana tabacum_A0A1S3XP18 LVKWLRNVMTSEDPKRAIDPKLIGTGFEEQMLLVLKIACFCTLENPKERPNSKDVRCMLTQIKH------
Nicotiana sylvestris_A0A1U7WH59 LVKWLKNVMTSEDPKRAIDPKLIGTGFEEQMLLVLKIACFCTLENPKERPNSKDVRCMLTQIKH------
Nicotiana attenuata_A0A1J6INR1 LVKWLRNVMTSEDPKRAIDQKLIGNGFEEQMLLVLKIACFCTLENPKERPNSKDVRCMLTQIKH------
Nicotiana tabacum_A0A1S3ZD83 LVKWLRNVMTSEDPKRAIDPKLIGSGFEEQMLLVLKIACFCTLENPKERPNSKDVRCMLTQIKH------
Solanum tuberosum_M1CKT4 LVKWLRNVMTSDDPKIAIDPKLRGNGYEEQMLLVLKIACFCTLDNPKERPNSKDVRCMLTQIKH------
Capsicum chinense_A0A2G3BFP7 LVKWLRNVMTSDDPKRAIDPNLIGNGYEEQMLLVLKIACFCTMDNPKERPNSKDVRCMLTQIKH------
Capsicum baccatum_A0A2G2WM84 LVKWLRNVMTSDDPKRAIDPNLMGNGYEEQMLLVLKIACFCTMDNPKERPNSKDVRCMLTQIKH------
Capsicum annuum_A0A2G2Y0L5 LVKWLRNVMTSDDPKRAIDPNLIGNGYEEQMLLVLKIACFCTMDNPKERPNSKDVRCMLTQIKH------
Solanum tuberosum_M1B7X0 LVKWMRNVMTSEDPNRAIDPKLMGNGNEDQMLLVLKIACFCTLENPKERPNSKDVRCMLMQIKH------
Capsicum chinense_A0A2G3CCQ8 LVKWMRNVMTSEDPNRAIDPKLMGNGNEEQMLLVLKIACFCTLENPKERPDSKDVRCMLMQIKH------
Capsicum annuum_A0A1U8G6T0 LVKWMRNVMTSEDPNRAIDPKLMGNGNEEQMLLVLKIACFCTLENPKERPNSKEVRCMLMQIKH------
Capsicum baccatum_A0A2G2X8C5 LVKWMRNVMTSEDPNRAIDPKLMGNGNEEQMLLVLKIACFCTLENPKERPNSKEARCMLMQIKH------
Nicotiana attenuata_A0A314KS23 LVKWMRNVMTSENPKRAIDPKLMGNGYEEQMLLVLKIACFCTLDNPKERPNSKDVRCMLTQIKP------
Coffea arabica_A0A6P6X9H5 LVHWMRNVMTSEDPKRAIDPKLLGNGYEEQMLLVLKIACFCTLENPKERPNSIDIRAMLFQIKYEKRQVM
Coffea arabica_A0A6P6X153 LVLWMRNVMTSEDPKRAIDPKLLGNGYEEQMLLVLKIACFCTLENPKERPNSIDIRAMLFQIKYEKR---
Coffea canephora_A0A068V365 LVLWMRNVMTSEDPKRAIDPKLLGNGYEEQMLLVLKIACFCTLENPKERPNSIDIRAMLFQIKYEKR---
Sesamum indicum_A0A6I9SRZ7 LVKWMRNVMTSEDPKRAIDPKLLGNGYEEQMLLVLKVACFCTLDNPKERPNSKDARCMLSQIRH------
Olea europaea subsp. europaea_A0A8S0R730 LVKWMRNVMTSEDPKRAIDPRLLGNGYEEQILLVLKIACFCTLDNPKERPDSKEIRCMLTQIKH------
Camellia sinensis var. sinensis_A0A4S4DBV0 LVKWMRNVMTSEDPTRAIDPRLMGNGDHENMLLVLKIACFCTLDDPKQRPNSKDVRCMLSQIKN------
Rhododendron simsii_A0A834L953 LVKWMRNVMTSEDPNRAIDRKLIGNGHEEKMLLVLKIACFCTLEDPKQRPNSKDVRCMLDQITH------
Vitis vinifera_F6GTQ3 LVKWMANIRTSDDPSRAIDRKLMGNGFEEQMLLVLKIACFCTVDDAKIRPNSKDVRTMLSQIKH------

Supplementary Figure 5. Alignment of the protein sequences of the SOBIR1 kinase domain from various plant species. Amino acid sequences of the SOBIR1 homologs from various plant species were obtained from the UniProt database (https://www.uniprot.org/). The alignment was visualized using JalView and only the amino acid sequences of the kinase domain are shown, with all the Tyr (Y) residues highlighted in green. The Tyr residues that are subject of this study are marked with arrowheads on the top, and their position in *Nb*SOBIR1 is indicated on the top. The RD motif (in which a conserved arginine (R) precedes the highly conserved catalytic aspartate (D)), present in all SOBIR1 kinase domains, is indicated by a box.

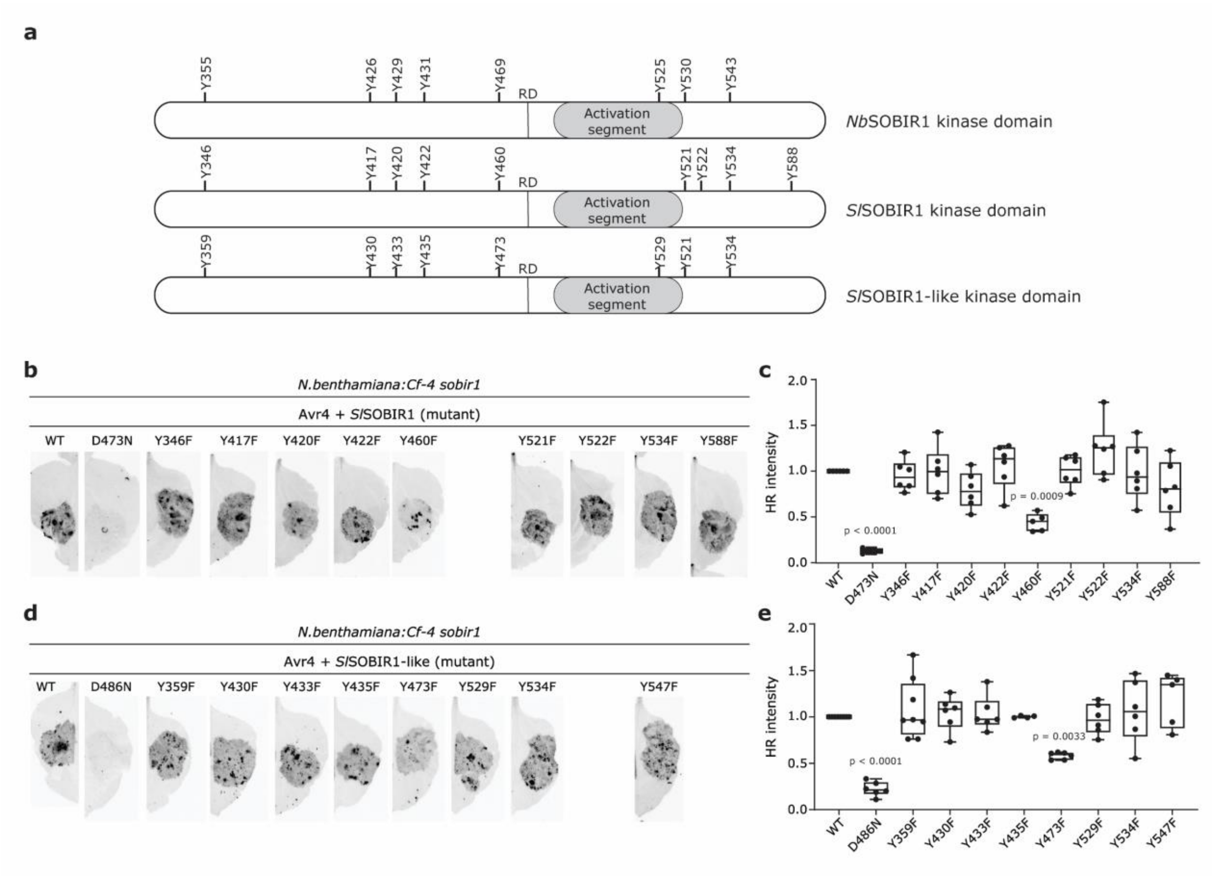

Supplementary Figure 6. *Sl*SOBIR1 Tyr460 and *Sl*SOBIR1-like Tyr473, which are the analogous residues of *Nb*SOBIR1 Tyr469, are essential for the Avr4/Cf-4-triggered HR in *N. benthamiana*. a, Schematic diagrams of the kinase domains of *Nb*SOBIR1, *Sl*SOBIR1 and *Sl*SOBIR1-like, with the location of the activation segment, the RD motif, and all Tyr residues indicated. **b**-**e**, Mutagenesis screen of all putative Tyr phosphorylation sites in *Sl*SOBIR1 (**b** and **c**) and *Sl*SOBIR1-like (**d** and **e**), as shown in Figure 2 for *Nb*SOBIR1, to determine their importance in immune signalling by complementation. Statistical analysis was performed with an ANOVA/Dunnett's multiple comparison test, compared with their corresponding WT. Dots indicate individual values (centre line, median; error bar, minima and maxima; n=6). Experiments were repeated at least three times, with similar results and representative pictures are shown. Source data are provided as a Source Data file.

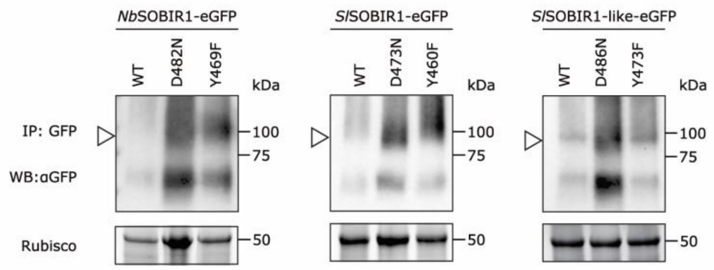

Supplementary Figure 7. Accumulation levels of *Nb*SOBIR1 Y469F, *Sl*SOBIR1 Y460F and *Sl*SOBIR1-like Y473F, as well as their corresponding WT and kinase-dead (D to N) versions, *in planta*. *Nb*SOBIR1 Y469F, *Sl*SOBIR1 Y460F and *Sl*SOBIR1-like Y473F, which fail to fully restore the Avr4-triggered HR in *N. benthamiana:Cf-4 sobir1* knock-out plants, were transiently expressed in *N. benthamiana:Cf-4 sobir1* knock-out plants in combination with Avr4 (both at an OD_{600} of 0.8), next to their respective WT that was combined with Avr4 as a positive control, and their kinase-dead D to N version that was combined with Avr4 as a negative control. Leaf samples were collected at 2 dpi and total protein extracts were subjected to IP of the GFP-tagged SOBIR1 mutants using GFP-affinity beads, followed by WB with αGFP antibody (upper panels). The amount of total protein that was used for the IP is reflected by the Rubisco band present in the stain-free gel (lower panels). Arrowheads indicate the band representing SOBIR1-eGFP. Note that transient expression of SOBIR1 WT in combination with Avr4 triggers an HR in *N. benthamiana:Cf-4 sobir1* plants, which explains the low accumulation levels of SOBIR1 WT.

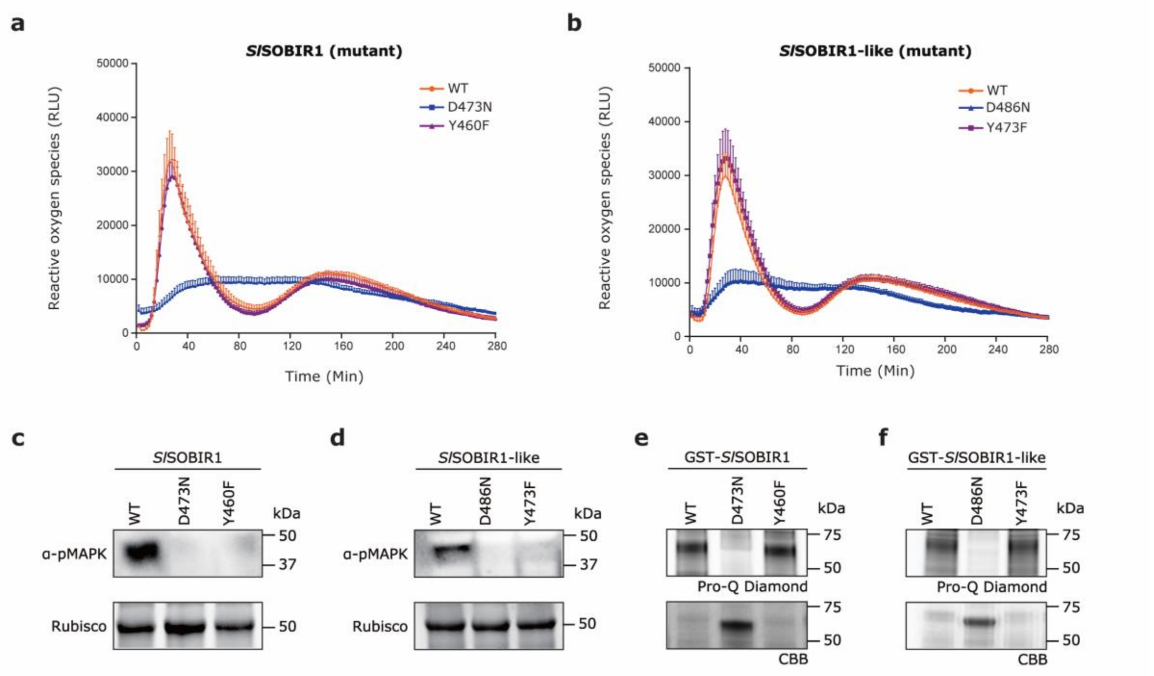

Supplementary Figure 8. *Sl*SOBIR1 Tyr460 and *Sl*SOBIR1-like Tyr473 are required for Avr4/Cf-4-induced MAPK activation, but not for ROS accumulation and their intrinsic kinase activity. a,**b**, The different Tyr mutants of *Sl*SOBIR1 and *Sl*SOBIR1-like were transiently expressed in leaves of the *N. benthamiana:Cf-4 sobir1* knock-out line, with their corresponding WTs as positive controls and kinase-dead mutants as negative controls. Leaf discs were taken from these plants at 24 hours after agro-infiltration, followed by adding 0.1 μM Avr4 protein and measuring ROS accumulation over time. ROS production is expressed as RLUs, and the data are represented as mean + SEM. Similar to SOBIR1 WT, all tested SOBIR1 Tyr mutants restored the Avr4/Cf-4-triggered ROS production in this complementation study. Only the results for *Sl*SOBIR1 WT, Y460F and D473N are shown in (**a**), and results for *Sl*SOBIR1-like WT, Y473F and D486N are shown in (**b**). **c**, **d**, *Sl*SOBIR1 Y460F and *Sl*SOBIR1-like Y473F, as well as their corresponding WTs and kinase-dead mutants, were transiently co-expressed with Avr4 in leaves of *N. benthamiana:Cf-4 sobir1* knock-out plants. Leaf samples were collected at 2 dpi and total protein extracts were subjected to immunoblotting with a p42/p44-erk antibody to determine the activation of downstream MAPKs by phosphorylation. **e**, **f**, The N-terminally GST-tagged cytoplasmic kinase domains of *Sl*SOBIR1 Y460F and *Sl*SOBIR1-like Y473F were produced in *E. coli*, with their corresponding WTs as positive controls and their kinase-dead mutants as negative controls. After SDS-PAGE of the *E. coli* lysates, the recombinant proteins were stained with Coomassie brilliant blue (lower panels), whereas the phosphorylation status of the kinase domains was determined by performing a Pro-Q Diamond stain (upper panels). Experiments were repeated at least three times and similar results were obtained. Representative pictures are shown. Source data are provided as a Source Data file.

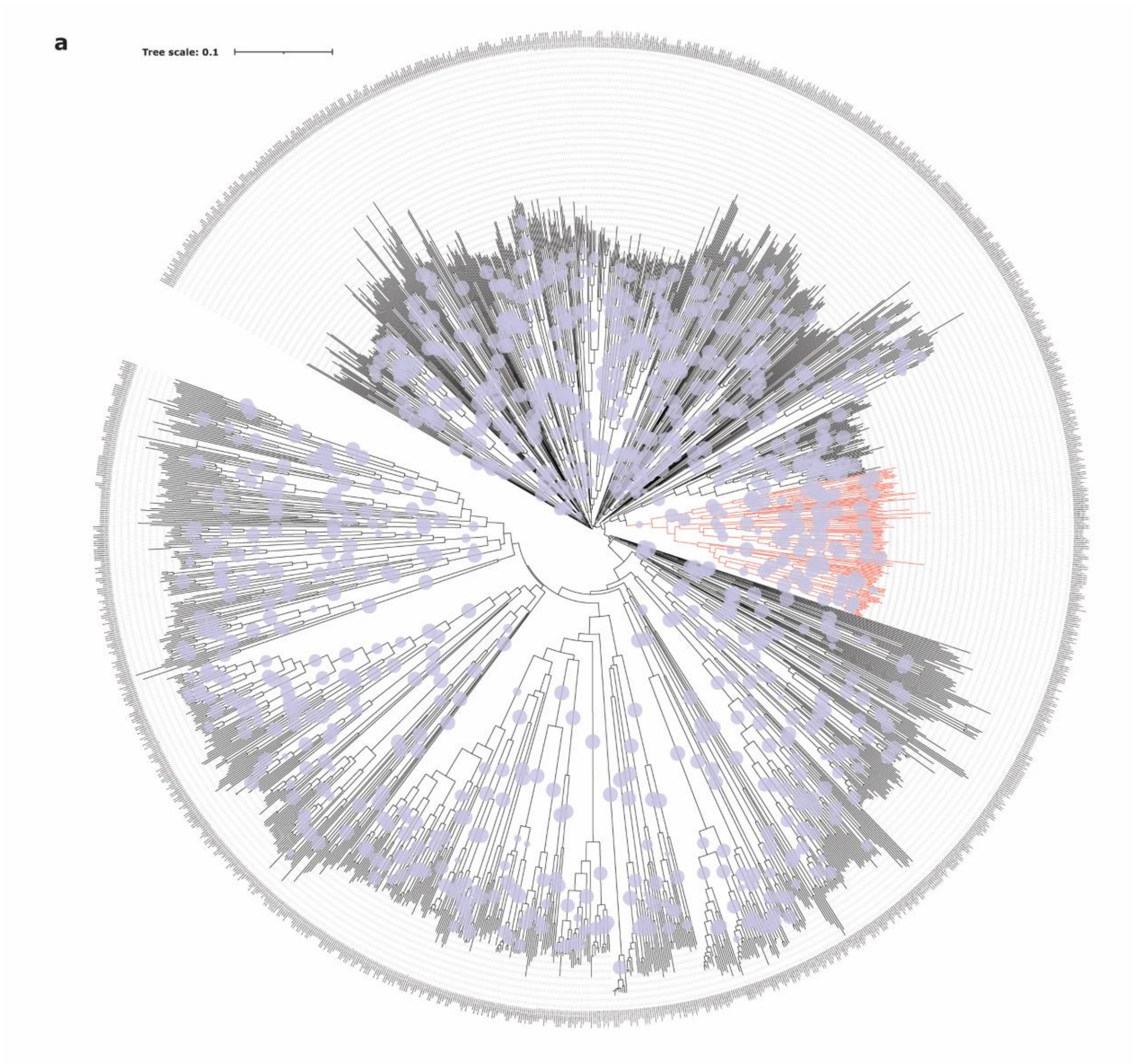
a
Tree scale: 0.1

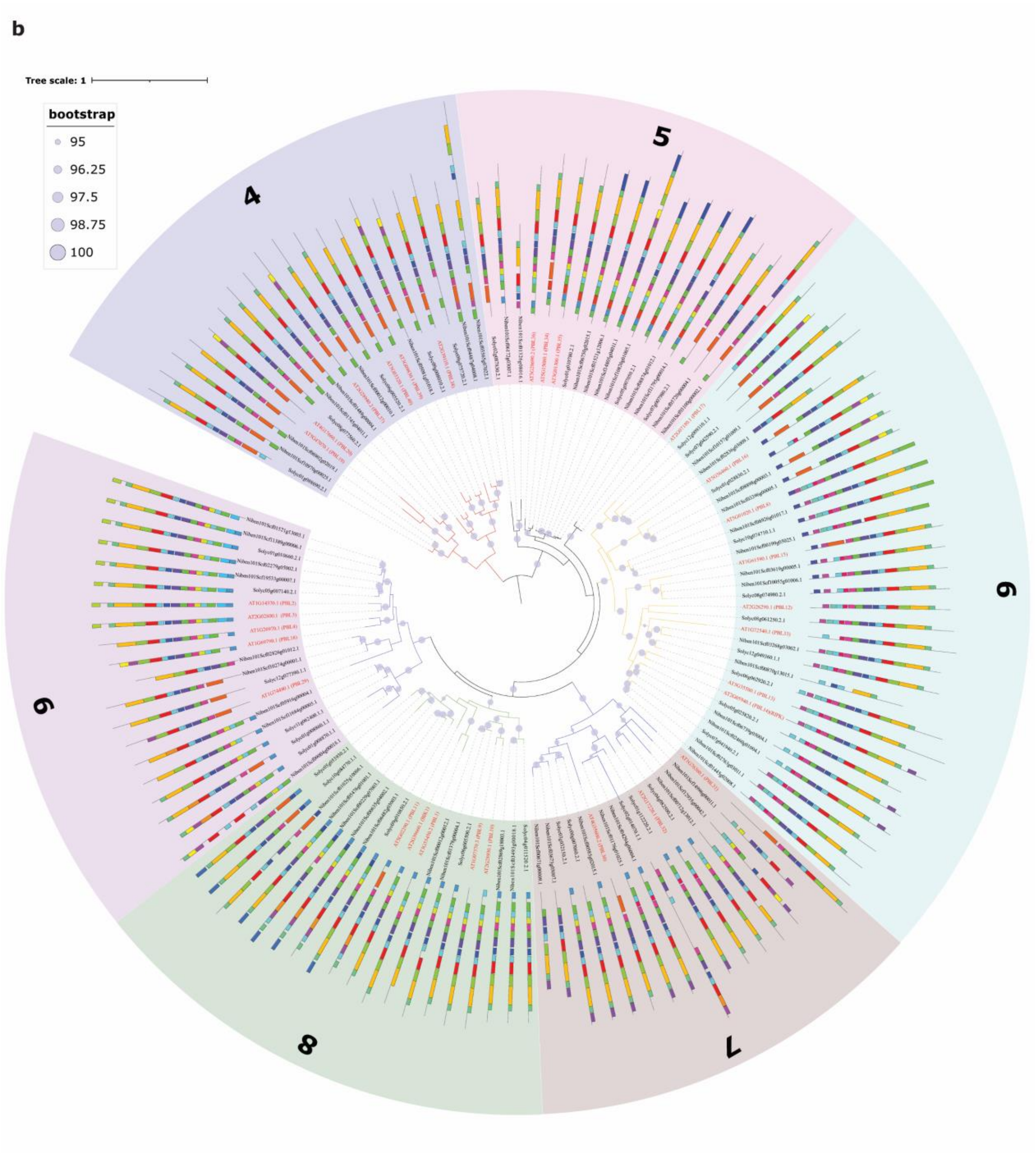

Supplementary Figure 9. Phylogenetic analysis of BIK1 homologs from Arabidopsis, tomato, and *N. benthamiana*. a, The amino acid sequences of only the kinase domain were extracted from all RLCK members from Arabidopsis, tomato, and *N. benthamiana* and aligned to subsequently generate a neighbour-joining phylogenetic tree, using QuickTree (Howe et al., 2002). The sub-clade of putative BIK1 homologs, which comprises 123 sequences, including *At*BIK1 (bootstrap support higher than 90%) is shown in red. **b**, Phylogenetic analysis of the RLCK-VII subfamily members from Arabidopsis (which are indicated in red), tomato, and *N. benthamiana*. Amino acid motifs identified in the complete protein sequence using MEME are shown (Bailey et al., 2009). All the members present in this tree were further assigned to six subfamilies, which are depicted in different colours. These subfamilies are referred to as subfamily 4, 5, 6, 7, 8, and 9, according to the RLCK-VII subfamilies in Arabidopsis reported previously by Rao et al. (2018).

a

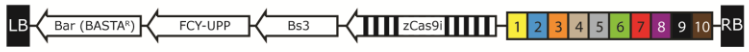
LB
Bar (BASTA^R)
FCY-UPP
Bs3
zCas9i
1
2
3
4
5
6
7
8
9
10
RB

b

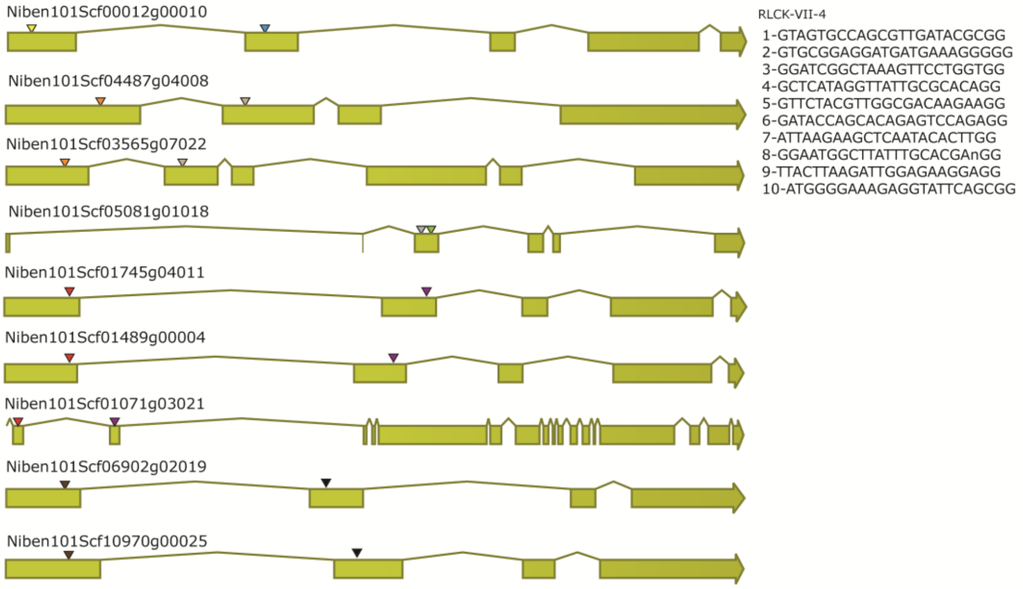
Niben101Scf00012g00010
Niben101Scf04487g04008
Niben101Scf03565g07022
Niben101Scf05081g01018
Niben101Scf01745g04011
Niben101Scf01489g00004
Niben101Scf01071g03021
Niben101Scf06902g02019
Niben101Scf10970g00025
RLCK-VII-4
1-GTAGTGCCAGCGTTGATACGCGG
2-GTGCGGAGGATGATGAAAGGGGG
3-GGATCGGCTAAAGTTCCTGGTGG
4-GCTCATAGGTTATTGCGCACAGG
5-GTTCTACGTTGGCGACAAGAAGG
6-GATACCAGCACAGAGTCCAGAGG
7-ATTAAGAAGCTCAATACACTTGG
8-GGAATGGCTTATTTGCACGAnGG
9-TTACTTAAGATTGGAGAAGGAGG
10-ATGGGGAAAGAGGTATTCAGCGG

c

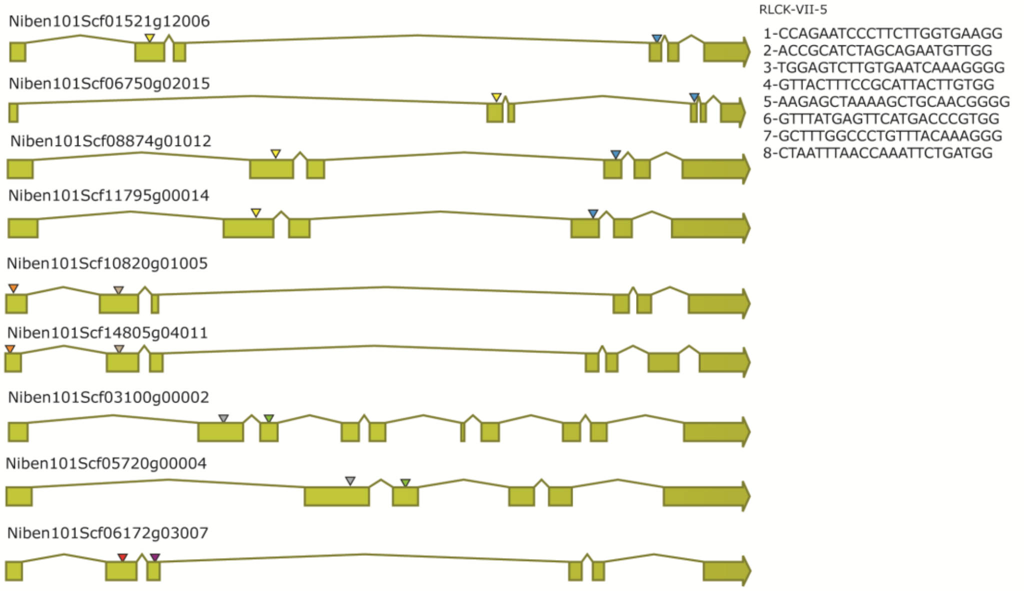
Niben101Scf01521g12006
Niben101Scf06750g02015
Niben101Scf08874g01012
Niben101Scf11795g00014
Niben101Scf10820g01005
Niben101Scf14805g04011
Niben101Scf03100g00002
Niben101Scf05720g00004
Niben101Scf06172g03007
RLCK-VII-5
1-CCAGAATCCCTTCTTGGTGAAGG
2-ACCGCATCTAGCAGAATGTTGG
3-TGGAGTCTTGTGAATCAAAGGGG
4-GTTACTTTCCGCATTACTTGTGG
5-AAGAGCTAAAAGCTGCAACGGGG
6-GTTTATGAGTTCATGACCCGTGG
7-GCTTTGGCCCTGTTTACAAAGGG
8-CTAATTTAACCAAATTCTGATGG

d

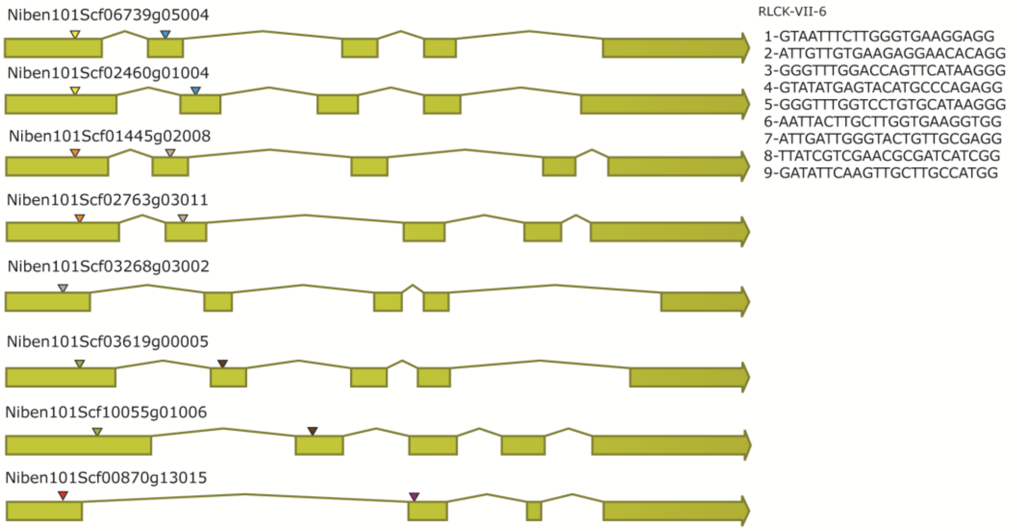
Niben101Scf06739g05004
Niben101Scf02460g01004
Niben101Scf01445g02008
Niben101Scf02763g03011
Niben101Scf03268g03002
Niben101Scf03619g00005
Niben101Scf10055g01006
Niben101Scf00870g13015
RLCK-VII-6
1-GTAATTTCTTGGGTGAAGGAGG
2-ATTGTTGTGAAGAGGAACACAGG
3-GGGTTTGGACCAGTTCATAAGGG
4-GTATATGAGTACATGCCCAGAGG
5-GGGTTTGGTCCTGTGCATAAGGG
6-AATTACTTGCTTGGTGAAGGTGG
7-ATTGATTGGGTACTGTTGCGAGG
8-TTATCGTCGAACGCGATCATCGG
9-GATATTCAAGTTGCTTGCCATGG

e

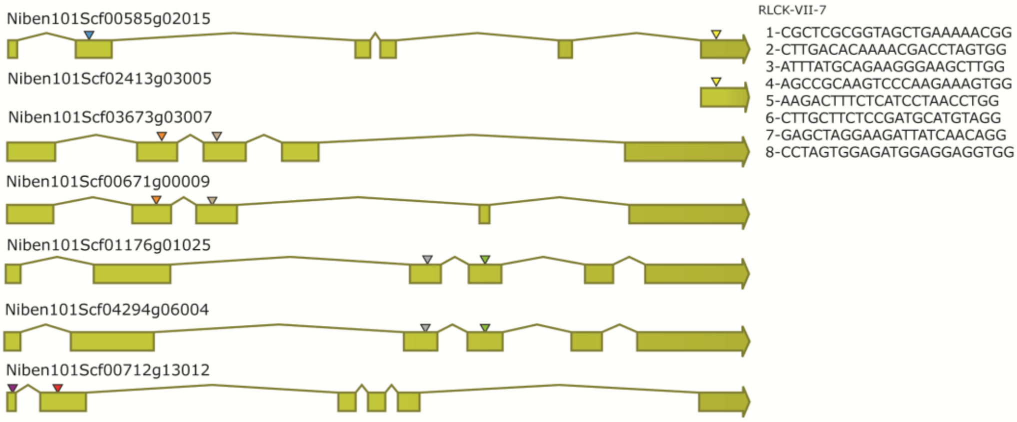
Niben101Scf00585g02015
Niben101Scf02413g03005
Niben101Scf03673g03007
Niben101Scf00671g00009
Niben101Scf01176g01025
Niben101Scf04294g06004
Niben101Scf00712g13012
RLCK-VII-7
1-CGCTCGCGGTAGCTGAAAAACGG
2-CTTGACACAAAACGACCTAGTGG
3-ATTTATGCAGAAGGGAAGCTTGG
4-AGCCGCAAGTCCCAAGAAAGTGG
5-AAGACTTTCTCATCCTAACCTGG
6-CTTGCTTCTCCGATGCATGTAGG
7-GAGCTAGGAAGATTATCAACAGG
8-CCTAGTGGAGATGGAGGAGGTGG

f

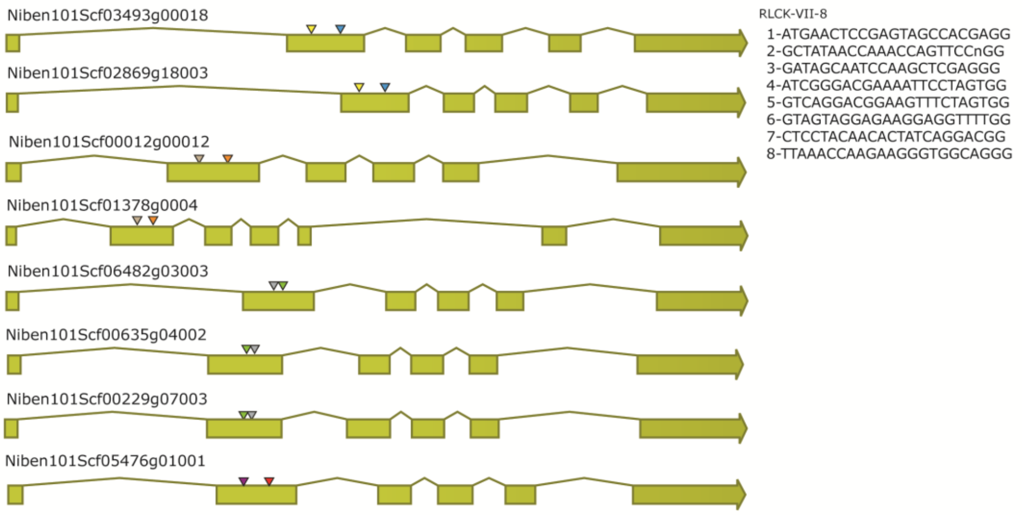

g

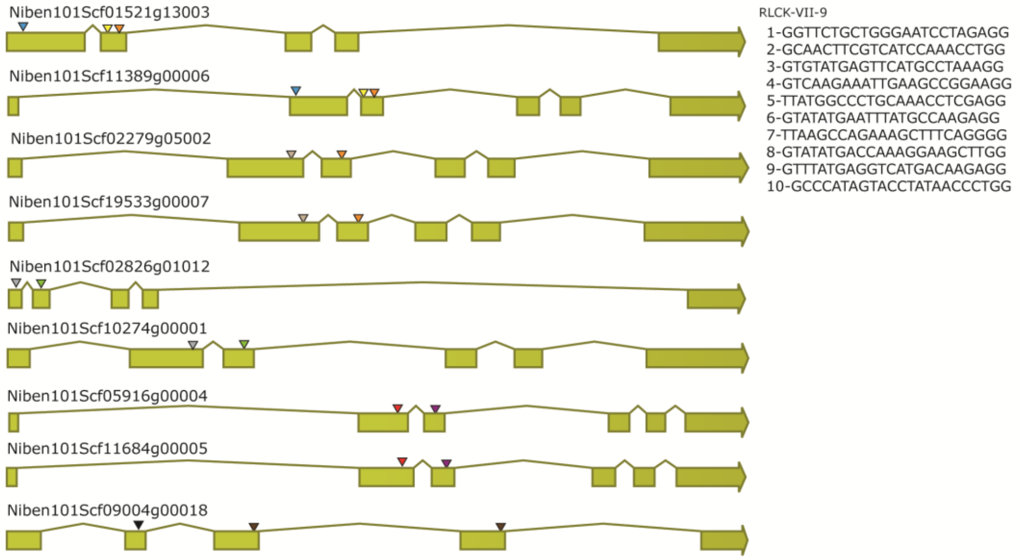

Supplementary Figure 10. Editing genes of RLCK-VII subfamilies in *N. benthamiana:Cf-4*. **a**, Scheme of the binary vector used for editing RLCK-VII genes. T-DNAs encompass the *Bar* gene (resistance to phosphinothricin; under control of the *nos* promoter), the FCY-UPP genes (p*AtUbq10* control)[78], the *Bs3* gene from pepper (*Capsicum annuum*; own promoter), the intron-optimized *zCas9i* gene (*AtRPS5a* promoter control) and up to 10 sgRNA transcriptional units driven by an *At*U6-26 promoter fragment. **b**-**g**, Gene models of *RLCK-VII* genes according to the solgenomics.net *N. benthamiana* genome release v1.01, with target sites indicated (coloured triangles). The colour code used for cut sites corresponds to the sgRNA array indicated in panel **a**. The target sites of the various sgRNAs are indicated for each subfamily next to the gene models.

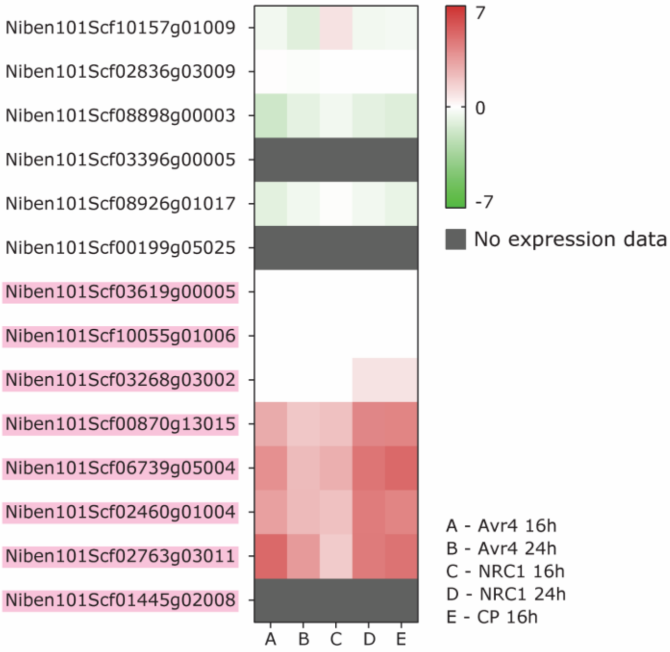

Supplementary Figure 11. Gene expression patterns of the 14 members from *N. benthamiana* RLCK-VII-6. Heat map of the relative expression (log2) of all 14 RLCK members of subfamily 6, as determined upon transient expression of Avr4, constitutively active NRC1 (Gabriëls et al., 2007), or the coat protein (CP) of potato virus X (Tameling et al., 2010), in leaves of *N. benthamiana* plants transgenic for both *Cf-4* and *Rx*, with the latter gene mediating recognition of the CP of PVX. The RLCK-encoding genes that were selected to be knocked out are highlighted in pink. NRC1, NB-LRR PROTEIN REQUIRED FOR HR-ASSOCIATED CELL DEATH 1; CP, coat protein.

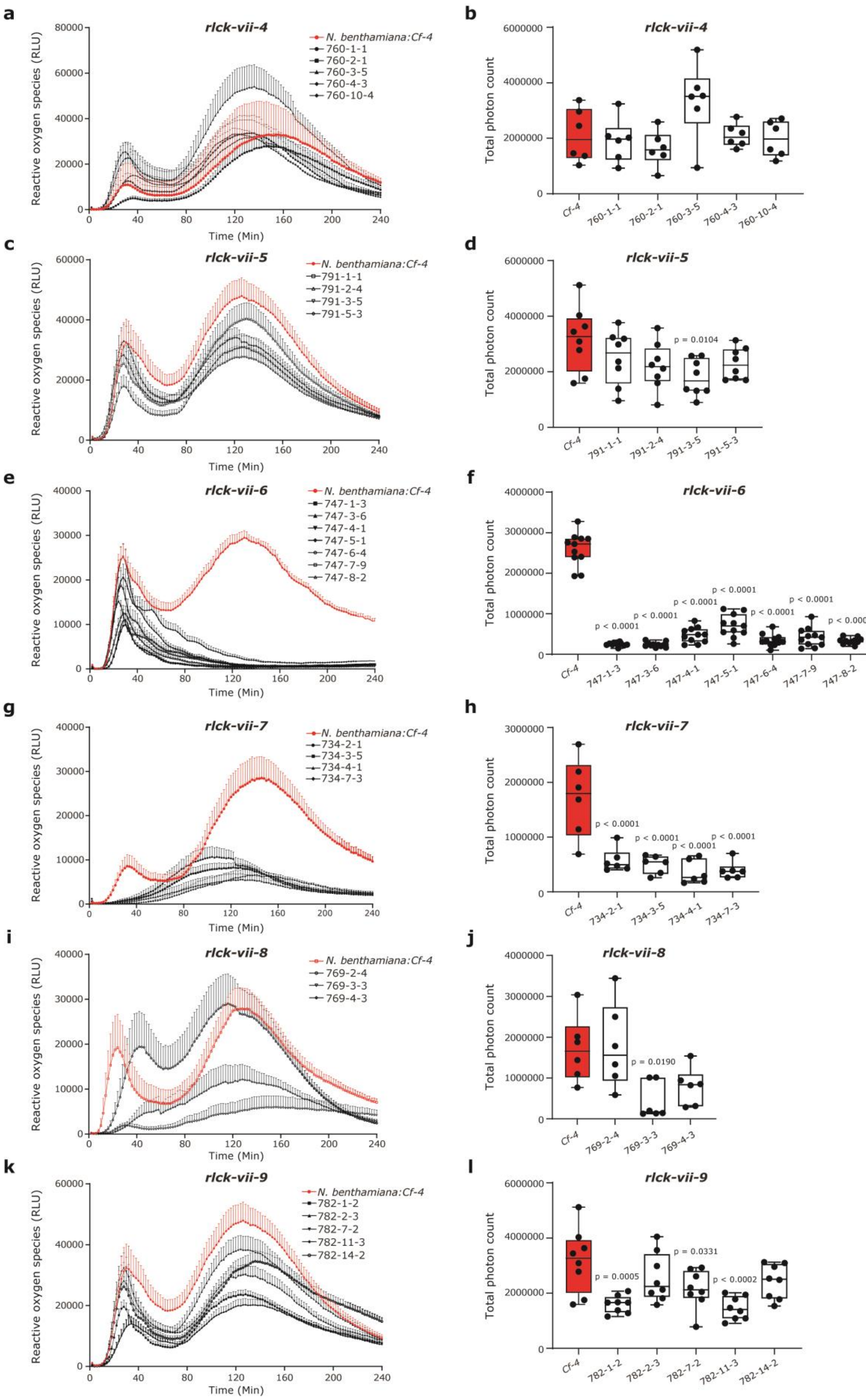
a
rlck-vii-4
N. benthamiana:Cf-4
760-1-1
760-2-1
760-3-5
760-4-3
760-10-4
Reactive oxygen species (RLU)
Time (Min)
b
rlck-vii-4
Total photon count
Cf-4
c
rlck-vii-5
N. benthamiana:Cf-4
791-1-1
791-2-4
791-3-5
791-5-3
d
rlck-vii-5
p = 0.0104
e
rlck-vii-6
N. benthamiana:Cf-4
747-1-3
747-3-6
747-4-1
747-5-1
747-6-4
747-7-9
747-8-2
f
rlck-vii-6
p < 0.0001
g
rlck-vii-7
N. benthamiana:Cf-4
734-2-1
734-3-5
734-4-1
734-7-3
h
rlck-vii-7
p < 0.0001
i
rlck-vii-8
N. benthamiana:Cf-4
769-2-4
769-3-3
769-4-3
j
rlck-vii-8
p = 0.0190
k
rlck-vii-9
N. benthamiana:Cf-4
782-1-2
782-2-3
782-7-2
782-11-3
782-14-2
l
rlck-vii-9
p = 0.0005
p = 0.0331
p < 0.0002

Supplementary Figure 12. Various subfamilies from RLCK-VII play an important role in the Avr4/Cf-4/SOBIR1-triggered ROS burst in *N. benthamiana:Cf-4*. Selected members from each RLCK-VII subfamily, being subfamilies 4, 5, 6, 7, 8 and 9, were targeted for knock-out in *N. benthamiana:Cf-4* by multiplex CRISPR/Cas technology. Subsequently, ROS accumulation induced upon Avr4 protein treatment of leaf discs obtained from five individual *rlck-vii-4* knock-out transformants (**a** and **b**), four *rlck-vii-5* knock-out transformants (**c** and **d**), seven *rlck-vii-6* knock-out transformants (**e** and **f**), four *rlck-vii-7* knock-out transformants (**g** and **h**), three *rlck-vii-8* knock-out transformants (**i** and **j**) and five *rlck-vii-9* knock-out transformants (**k** and **l**), was determined. For this, leaf discs were treated with 0.1 µM Avr4 protein, and the generation of ROS was monitored. Note that all *rlck* knock-out transformants were tested in the T1 generation. (**a**, **c**, **e**, **g**, **i**, and **k**) ROS production is expressed as RLUs, and the data are represented as mean + SEM (n=8). (**b**, **d**, **f**, **h**, **j**, and **l**) Statistical analysis was performed with an ANOVA/Dunnett's multiple comparison test, compared with their corresponding WT. Dots indicate individual values (centre line, median; error bar, minima and maxima). The ROS profiles of the positive control (*N. benthamiana:Cf-4*), included in all assays, are indicated in red in all the line charts. Similar results were obtained in three replicates and data from one representative experiment are shown. Source data are provided as a Source Data file.

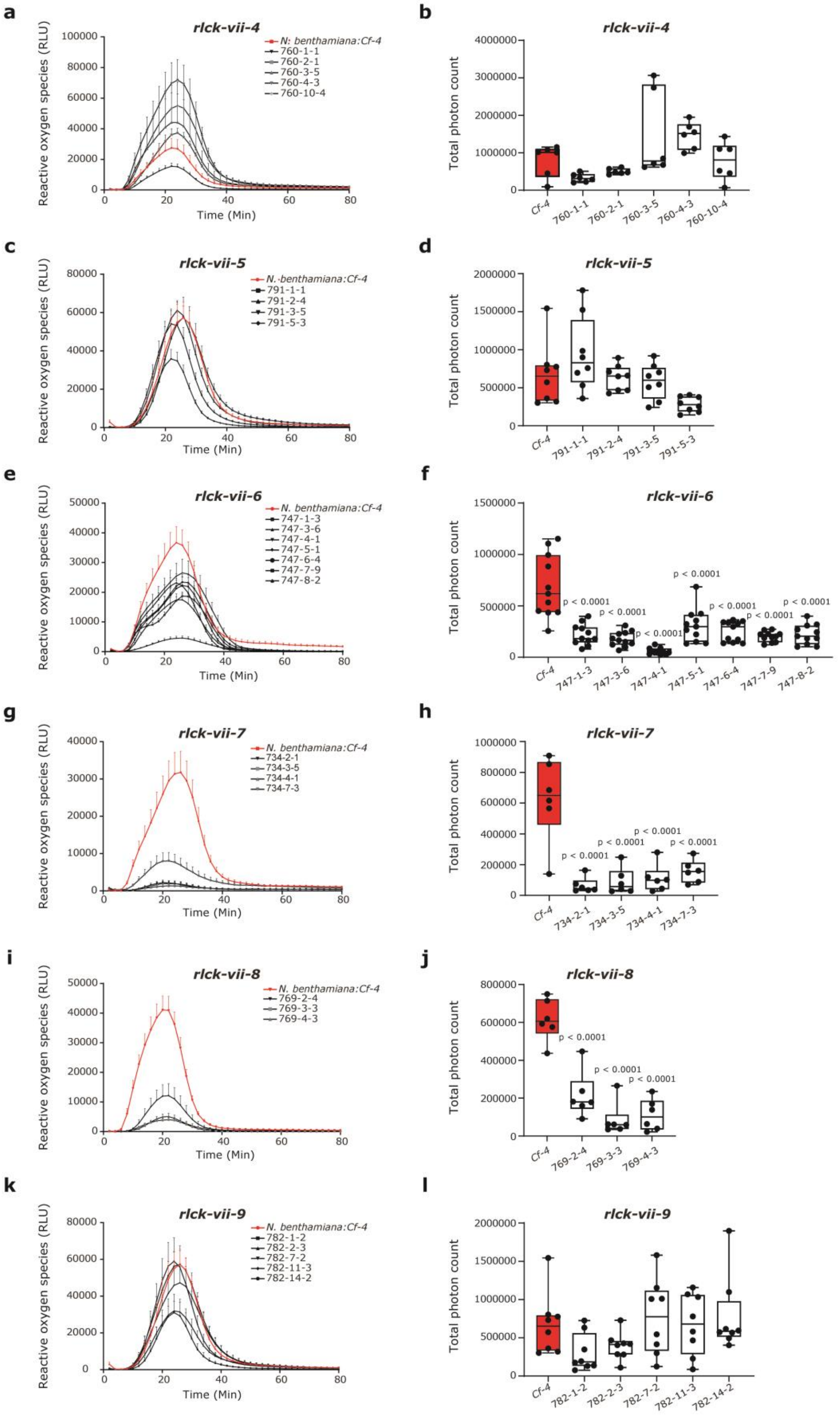
a
rlck-vii-4
Reactive oxygen species (RLU)
Time (Min)
N. benthamiana:Cf-4
760-1-1
760-2-1
760-3-5
760-4-3
760-10-4
b
rlck-vii-4
Total photon count
Cf-4
760-1-1
760-2-1
760-3-5
760-4-3
760-10-4
c
rlck-vii-5
Reactive oxygen species (RLU)
Time (Min)
N. benthamiana:Cf-4
791-1-1
791-2-4
791-3-5
791-5-3
d
rlck-vii-5
Total photon count
Cf-4
791-1-1
791-2-4
791-3-5
791-5-3
e
rlck-vii-6
Reactive oxygen species (RLU)
Time (Min)
N. benthamiana:Cf-4
747-1-3
747-3-6
747-4-1
747-5-1
747-6-4
747-7-9
747-8-2
f
rlck-vii-6
Total photon count
p < 0.0001
Cf-4
747-1-3
747-3-6
747-4-1
747-5-1
747-6-4
747-7-9
747-8-2
g
rlck-vii-7
Reactive oxygen species (RLU)
Time (Min)
N. benthamiana:Cf-4
734-2-1
734-3-5
734-4-1
734-7-3
h
rlck-vii-7
Total photon count
p < 0.0001
Cf-4
734-2-1
734-3-5
734-4-1
734-7-3
i
rlck-vii-8
Reactive oxygen species (RLU)
Time (Min)
N. benthamiana:Cf-4
769-2-4
769-3-3
769-4-3
j
rlck-vii-8
Total photon count
p < 0.0001
Cf-4
769-2-4
769-3-3
769-4-3
k
rlck-vii-9
Reactive oxygen species (RLU)
Time (Min)
N. benthamiana:Cf-4
782-1-2
782-2-3
782-7-2
782-11-3
782-14-2
l
rlck-vii-9
Total photon count
Cf-4
782-1-2
782-2-3
782-7-2
782-11-3
782-14-2

Supplementary Figure 13. RLCK-VII-6, -7 and -8 from *N. benthamiana* also play a positive role in the flg22/FLS2-triggered ROS burst. ROS production, triggered upon treatment with flg22, by discs taken from leaves of *rlck-vii-4* (**a** and **b**), *rlck-vii-5* (**c** and **d**), *rlck-vii-6* (**e** and **f**), *rlck-vii-7* (**g** and **h**), *rlck-vii-8* (**i** and **j**) and *rlck-vii-9* (**k** and **l**) *N. benthamiana:Cf-4* knock-out plants from the T1 generation, was measured. For this, leaf discs were taken from the different knock-out plants, as well as from *N. benthamiana:Cf-4* (the positive control), followed by treatment with a final concentration of 0.1 μM flg22 peptide and subsequent monitoring of the accumulation of ROS. (**a**, **c**, **e**, **g**, **i**, and **k**) ROS production is expressed as RLUs, and the data are represented as mean + SEM (n=8). (**b**, **d**, **f**, **h**, **j**, and **l**) Statistical analysis was performed with an ANOVA/Dunnett's multiple comparison test, compared with their corresponding WT. Dots indicate individual values (centre line, median; error bar, minima and maxima). The ROS traces of the positive control are indicated in red in all the line charts. All experiments were repeated at least three times and data from one representative experiment are shown. Source data are provided as a Source Data file.

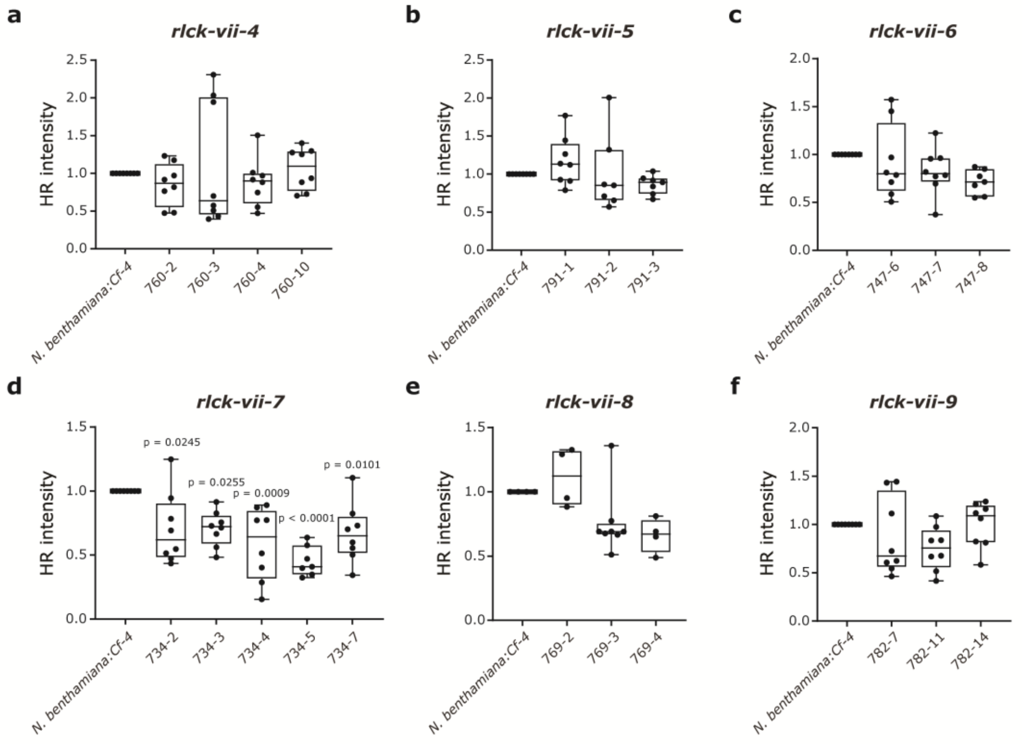

Supplementary Figure 14. RLCK-VII-7 is required for the Avr4/Cf-4-triggered HR in *N. benthamiana:Cf-4*. A solution of 5 μM pure Avr4 protein was infiltrated in leaves of the *N. benthamiana:Cf-4 rlck-vii-4* (**a**), *rlck-vii-5* (**b**), *rlck-vii-6* (**c**), *rlck-vii-7* (**d**), *rlck-vii-8* (**e**) and *rlck-vii-9* (**f**) transformants, and the Avr4/Cf-4-triggered HR was subsequently imaged using the ChemiDoc and quantified using Image Lab, at 2 dpi. All individual quantifications are shown as dots (n=8) and the means as lines. Statistical analysis was performed with an ANOVA/Dunnett's multiple comparison test, compared with *N. benthamiana:Cf-4.* Experiments were repeated at least three times with similar results, and representative results are shown. Source data are provided as a Source Data file.

a

	Niben101Scf03619g00005	Niben101Scf10055g01006	Niben101Scf03268g03002	Niben101Scf00870g13015	Niben101Scf06739g05004	Niben101Scf02460g01004	Niben101Scf02763g03011	Niben101Scf01445g02008
rlck-vii-6 #1	+1 bp; +1 bp	+1 bp; +1 bp	-3 bp	-12 bp; +1 bp	inversion and deletion	+1 bp; +1 bp	-5 bp; -5 bp	-1 bp; +1 bp/-1 bp
rlck-vii-6 #2	+1 bp; +1 bp	+1 bp; +1 bp	-3 bp	+1 bp; +1 bp	inversion and deletion	+1 bp; +1 bp	-5 bp; -5 bp	-1 bp; +1 bp/-1 bp
rlck-vii-6 #3	+1 bp; +1 bp	+1 bp; +1 bp	-1 bp	-10 bp; -2 bp	inversion, insertion and deletion	+1 bp; +1 bp	inversion and deletion	-14 bp; -4 bp

b

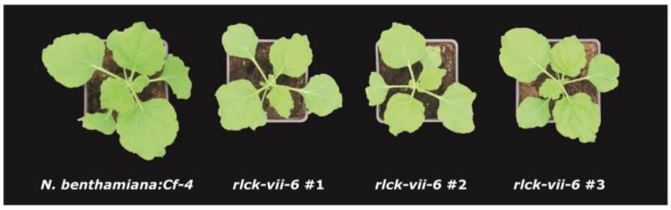

c

	Niben101Scf00712g13012	Niben101Scf04294g06004	Niben101Scf01176g01025	Niben101Scf00585g02015	Niben101Scf03673g03007	Niben101Scf00671g00009	Niben101Scf02413g03005
rlck-vii-7 #1	-7 bp; +1 bp	-4 bp; +1 bp	+1 bp; -1 bp	-1 bp; -1 bp	inversion, insertion and deletion	-1 bp; -1 bp	-1 bp
rlck-vii-7 #2	-8 bp; -2 bp	-3 bp; -3 bp	-7 bp; +1 bp	+1 bp; -9 bp	+1 bp; -6 bp	+1 bp; -9 bp	-9 bp

d

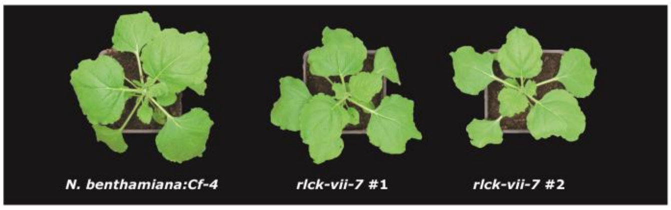

e

	Niben101Scf03493g00018	Niben101Scf02869g18003	Niben101Scf01378g00004	Niben101Scf00012g00012	Niben101Scf06482g03003	Niben101Scf00635g04002	Niben101Scf00229g07003	Niben101Scf05476g01001	Niben101Scf01025g10006
rlck-vii-8 #1	-; -5 bp	-94 bp; -4 bp	-7 bp; -7 bp	-11 bp; -8 bp	-5 bp; -1 bp	-4 bp; +2 bp	-4 bp; +2 bp	-11 bp; -8 bp	-7 bp; -7 bp
rlck-vii-8 #2	-; -5 bp	-94 bp; -4 bp	-7 bp; -7 bp	-11 bp; -8 bp	-5 bp; -1 bp	-6 bp; -5 bp	-4 bp; +2 bp	-11 bp; -8 bp	-7 bp; -7 bp

f

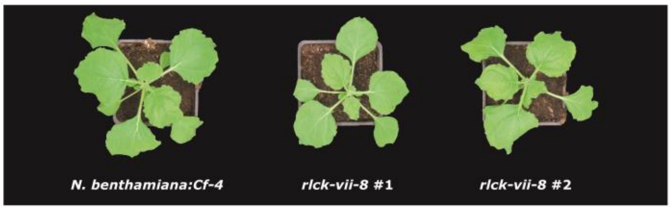

Supplementary Figure 15. Genotypes and morphological phenotypes of the different *rlck-vii* knock-out lines. Overview of the types of mutations present in all the *RLCK-VII-6* members (**a**),

RLCK-VII-7 members (**c**), and *RLCK-VII-8* members (**e**) in the independent homozygous *N. benthamiana:Cf-4* knock-out lines. Morphological phenotypes of *N. benthamiana:Cf-4* and the three independent *rlck-vii-6* (**b**), two independent *rlck-vii-7* (**d**), and two independent *rlck-vii-8* (**f**) knock-out lines. All plants were grown in soil under the same conditions and were photographed when they were four to five weeks old.

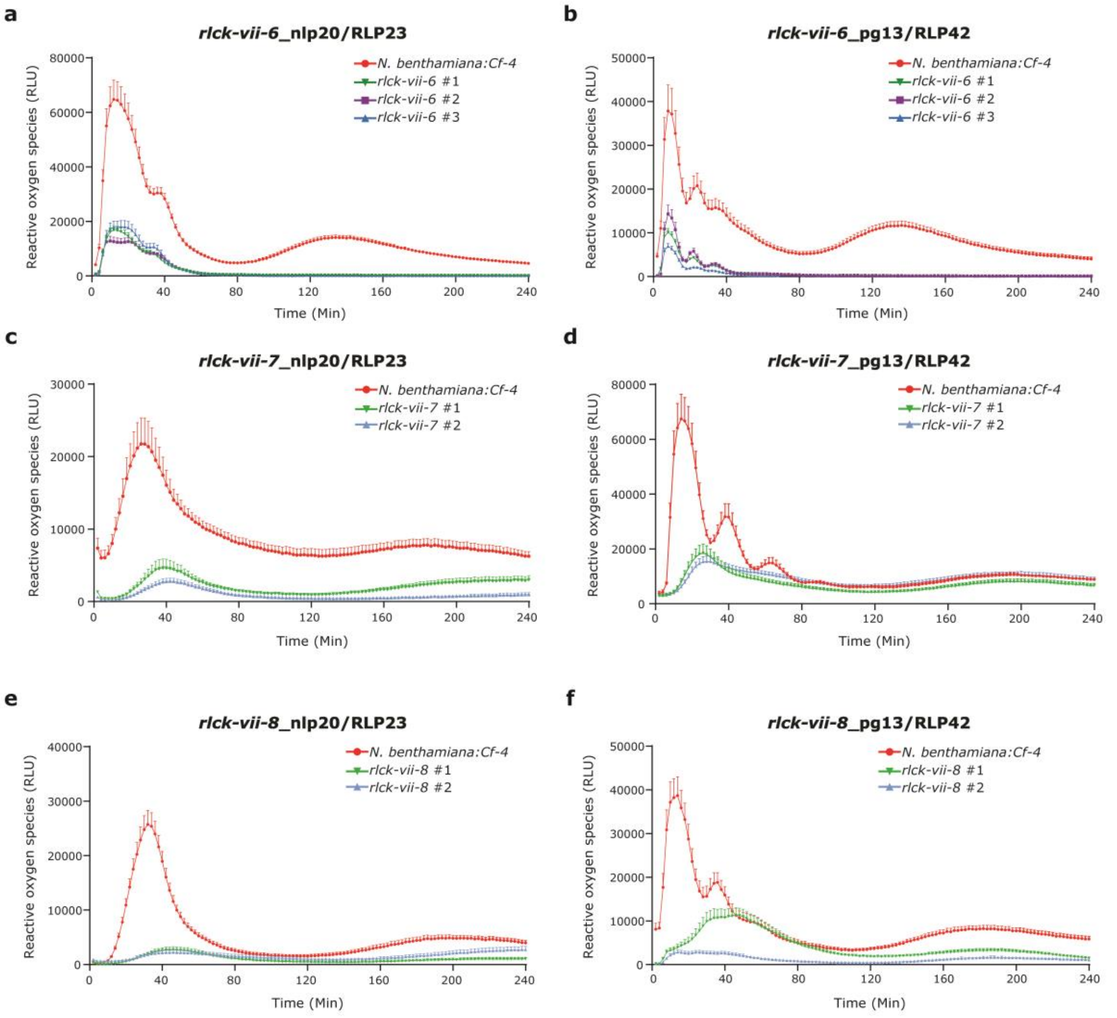

Supplementary Figure 16. Members of RLCK-VII-6, -7, and -8 differentially contribute to ROS accumulation in *N. benthamiana* induced by nlp20/RLP23 and pg13/RLP42 combinations. Leaf discs taken from independent *N. benthamiana:Cf-4 rlck-vii-6* (**a** and **b**), *rlck-vii-7* (**c** and **d**), and *rlck-vii-8* (**e** and **f**) lines, as well as from *N. benthamiana:Cf-4*, transiently expressing either RLP23 or RLP42, were treated with the corresponding elicitors at a 1 µM concentration and the accumulation of ROS was monitored. ROS production is expressed as RLUs, and the data are represented as mean + SEM (n=8). Source data are provided as a Source Data file.

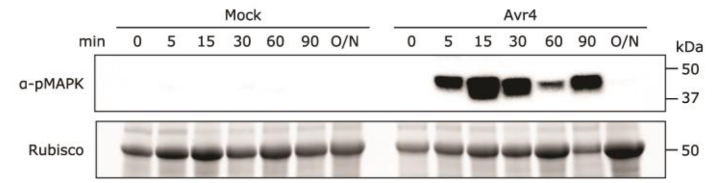

Supplementary Figure 17. The Avr4 protein triggers a swift MAPK activation in *N. benthamiana:Cf-4*. Water (mock) or 5 µM of pure Avr4 protein was infiltrated in leaves of *N. benthamiana:Cf-4*. Leaf samples were taken at the indicated time points after Avr4 infiltration, and total protein extracts were subjected to immunoblotting using a p42/p44-erk antibody specifically detecting MAPKs that are activated by phosphorylation (α-pMAPK). Rubisco is shown as a total protein loading control. O/N, overnight. The experiment was repeated two times with similar results and a representative result is shown.

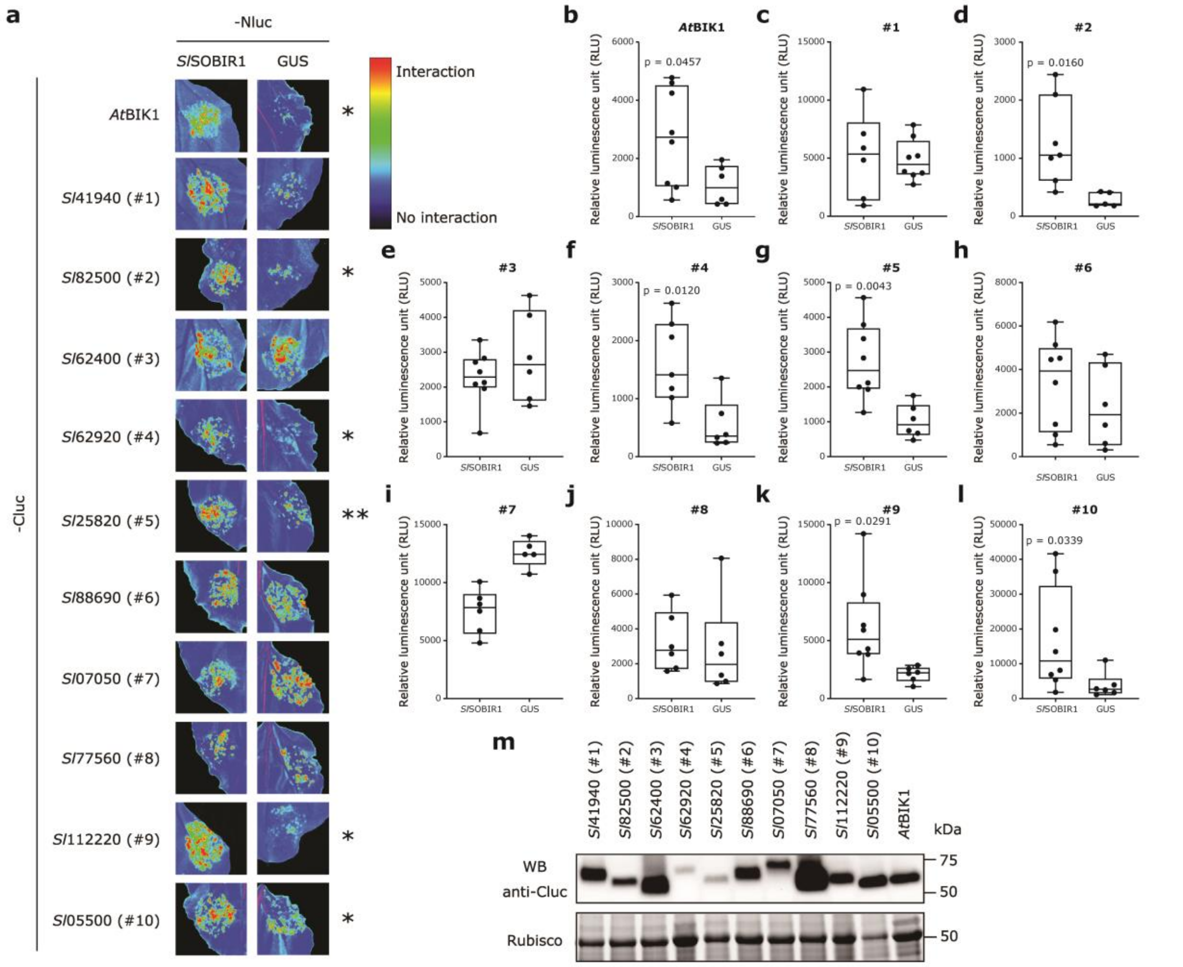

Supplementary Figure 18. A split-luciferase assay shows the interaction between several tomato RLCKs and *Sl*SOBIR1. a, Cluc-tagged Arabidopsis BIK1 (*At*BIK1) and tomato RLCKs and were transiently co-expressed with Nluc-tagged *Sl*SOBIR1. Nluc-tagged GUS was included as a negative control. At 3 dpi leaves were sprayed with luciferin and luciferase activity was subsequently observed using a ChemiDoc device. Representative images of two independent experiments, each including six leaves for each interaction that was tested, are shown. Blue indicates that there is no luciferase signal, and a colouration increasing to red indicates a stronger luciferase signal, representing interaction to take place between the proteins. **b**-**l**, Quantification of the luminescence as shown in **a**. Dots indicate individual values (centre line, median; error bar, minima and maxima, n=6) Statistical significance was determined by Student's t-tests. **m**, Accumulation levels of the various tomato RLCKs, fused to the C-terminal part of the luciferase enzyme (Cluc). At 3 dpi an immunoprecipitation assay, followed by immunoblotting using anti-luciferase antibody (α-Cluc) was performed. *Sl*41940, Solyc07G041940-Cluc; *Sl*82500, Solyc04G082500-Cluc; *Sl*62400, Solyc11G062400-Cluc; *Sl*62920, Solyc06G062920-Cluc; *Sl*25820, Solyc05G025820-Cluc; *Sl*88690, Solyc01G088690-Cluc; *Sl*07050, Solyc05G007050-Cluc; *Sl*77560, Solyc08G077560-Cluc; *Sl*112220, Solyc01G112220-Cluc; *Sl*05500, Solyc06G005500-Cluc. Source data are provided as a Source Data file.

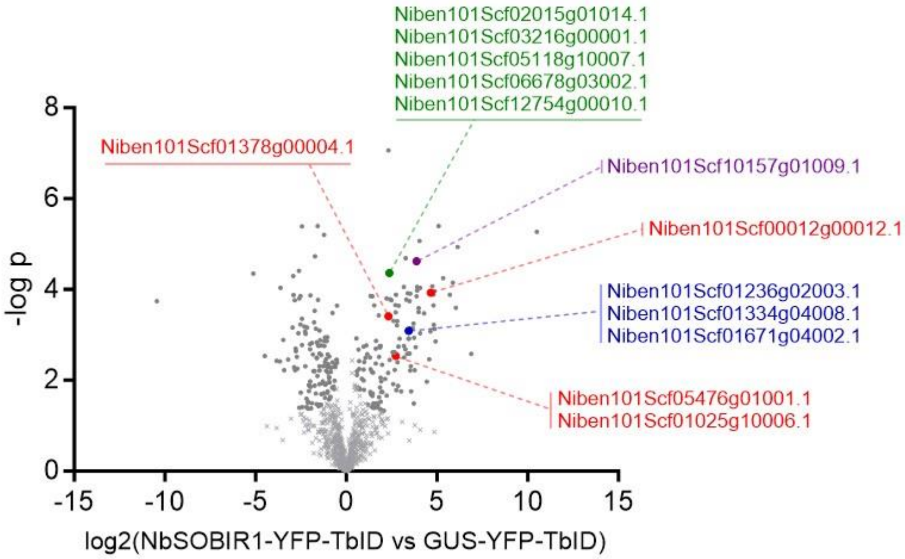

Supplementary Figure 19. Volcano plot showing the RLCKs that were identified by proximity-dependent labelling using *Nb*SOBIR1-YFP-TbID as a transiently expressed bait in *N. benthamiana*. The change from grey crosses to closed circles marks the threshold for statistical significance. The RLCKs that were significantly enriched in three independent experiments in which *Nb*SOBIR1-YFP-TbID (on the right) was used as a bait, when compared to GUS-YFP-TbID (on the left), are indicated with dots having different colours. Note that for RLCK-VII-6 (purple) and RLCK-VII-8 (red) we were able to identify individual RLCKs, with the exception of Niben101Scf05476g01001 being detected together with Niben101Scf01025g10006, which is its closest paralog in the phylogeny shown in Figure S9. For RLCK-IV (green) and RLCK-VIII (blue) the determined peptide sequences did not allow the assignment of individual RLCK members.

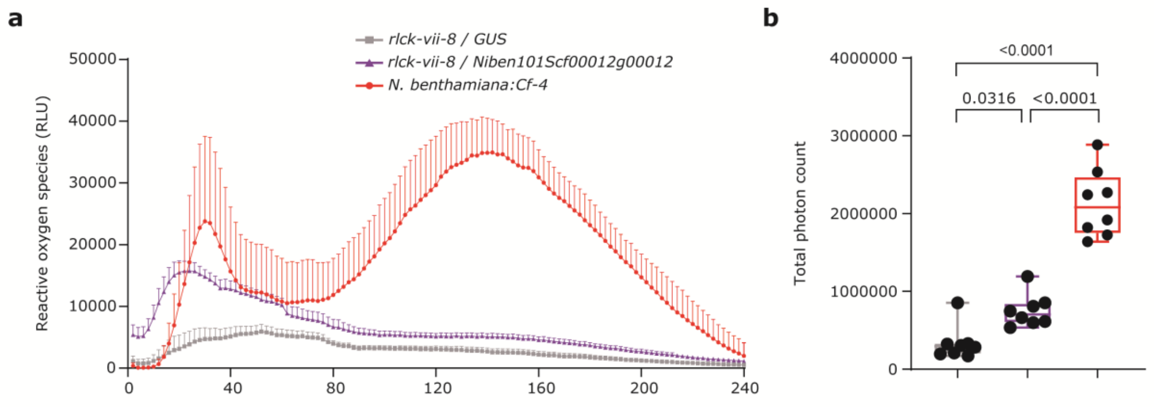

Supplementary Figure 20. A complementation assay shows that RLCK Niben101Scf00012g00012 partially restores the ROS burst. A complementation assay with the RLCK-VII-8 family member Niben101Scf00012g00012 (*Nb*00012) was performed by transient expression, driven by the 35S promotor, in the *rlck-vii-8* #1 knock-out line of *N. benthamiana:Cf-4*. **a**, ROS production over time upon treatment of leaf discs transiently expressing either *GUS* or *Nb00012* with Avr4, is expressed as RLUs and the data are represented as mean + SEM (n=8). *N. benthamiana:Cf-4* was taken along as a control. Note that only partial restoration of the biphasic ROS burst takes place. **b**, Total amount of ROS generated by the leaf discs, of which the profile is shown in **a**. Dots indicate individual values (centre line, median; error bar, minima and maxima, n=8) (One-way ANOVA with Tukey's test). The total photon count represents the sum of all the relative light units (RLUs) measured over a four-hour period. Letters indicate statistical groupings. Experiments were repeated three times with similar results. Source data are provided as a Source Data file.

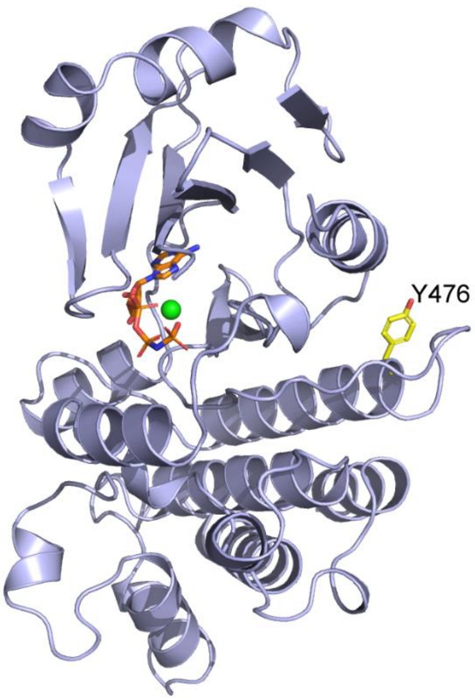

Supplementary Figure 21. The overall structure of the inactive *At*SOBIR1 kinase domain and position of the important Tyr residues. The ribbon diagram of the SOBIR1 kinase domain is coloured in light blue. The non-hydrolysable ATP analogue AMP-PNP and Mg^{2+} are presented as an orange stick and a green sphere, respectively. *At*SOBIR1 Tyr476, which is analogous to *Nb*SOBIR1 Tyr469, is indicated as a yellow stick.

Supplementary Table 1. ***RLCK*** **genes selected to be targeted by single guide (sg)RNAs.**

Subfamily	Gene name	Amount of sgRNAs	Target sequence (5′ - 3′)	Note
4	*Niben101Scf10970 g00025*	2	TTACTTAAGATTGGAGA AGGAGG; ATGGGGAAAGAGGTATT CAGCGG	
	Niben101Scf06902 g02019			
	Niben101Scf01745 g04011	3	ATTAAGAAGCTCAATAC ACTTGG; GGAATGGCTTATTTGCA CGACGG	
	Niben101Scf01489 g00004			
	Niben101Scf01071 g03021			Not present in the phylogeny, but clustered to this subfamily according to iTAK*
	Niben101Scf00012 g00010	2	GTAGTGCCAGCGTTGAT ACGCGG; GTGCGGAGGATGATGAA AGGGGG	
	Niben101Scf03565 g07022	2	GGATCGGCTAAAGTTCC TGGTGG; GCTCATAGGTTATTGCG CACAGG	
	Niben101Scf04487 g04008			
	Niben101Scf05081 g01018		GTTCTACGTTGGCGACA AGAAGG; GATACCAGCACAGAGTC CAGAGG	
5	*Niben101Scf06172 g03007*	2	GCTTTGGCCCTGTTTAC AAAGGG; CTAATTTAACCAAATTCT GATGG	
	Niben101Scf01326 g08016			Not expressed according to QUT
	Niben101Scf06750 g02015	2	CCAGAATCCCTTCTTGG TGAAGG; ACCGCATCTAGCAGAAT GTTGG	
	Niben101Scf01521 g12006			
	Niben101Scf08874 g01012			
	Niben101Scf11795 g00014			
	Niben101Scf14805 g04011	2	TGGAGTCTTGTGAATCA AAGGGG; GTTACTTTCCGCATTACT TGTGG	
	Niben101Scf10820 g01005			
	Niben101Scf05720 g00004	2	AAGAGCTAAAAGCTGCA ACGGGG;	

	Niben101Scf03100 g00002		GTTTATGAGTTCATGAC CCGTGG	
6	*Niben101Scf10157 g01009*			Not selected, see Figure S11
	Niben101Scf02836 g03009			Not selected, see Figure S11
	Niben101Scf08898 g00003			Not selected, see Figure S11
	Niben101Scf03396 g00005			Not selected, see Figure S11
	Niben101Scf08926 g01017			Not selected, see Figure S11
	Niben101Scf00199 g05025			Not selected, see Figure S11
	Niben101Scf03619 g00005	2	AATTACTTGCTTGGTGA AGGTGG; ATTGATTGGGTACTGTT GCGAGG	
	Niben101Scf10055 g01006			
	Niben101Scf03268 g03002	1	GGGTTTGGTCCTGTGCA TAAGGG	
	Niben101Scf00870 g13015	2	TTATCGTCGAACGCGAT CATCGG; GATATTCAAGTTGCTTG CCATGG	
	Niben101Scf06739 g05004	2	GTAATTTCTTGGGTGAA GGAGG; ATTGTTGTGAAGAGGAA CACAGG	
	Niben101Scf02460 g01004			
	Niben101Scf02763 g03011	2	GGGTTTGGACCAGTTCA TAAGGG; GTATATGAGTACATGCC CAGAGG	
	Niben101Scf01445 g02008			
7	*Niben101Scf14996 g00011*			Not expressed according to QUT
	Niben101Scf12935 g00042			Not expressed according to QUT
	Niben101Scf00712 g13012	2	GAGCTAGGAAGATTATC AACAGG; CCTAGTGGAGATGGAGG AGGTGG	
	Niben101Scf04294 g06004	2	AAGACTTTCTCATCCTAA CCTGG; CTTGCTTCTCCGATGCA TGTAGG	
	Niben101Scf01176 g01025			
	Niben101Scf00585 g02015	2	CGCTCGCGGTAGCTGAA AAACGG; CTTGACACAAAACGACC TAGTGG	

	Niben101Scf03673g03007	2	ATTTATGCAGAAGGGAAGCTTGG; AGCCGCAAGTCCCAAGAAAGTGG	
	Niben101Scf00671g00009			
	Niben101Scf02413g03005	1	CTTGACACAAAACGACCTAGTGG	Not present in the phylogeny, but clustered to this subfamily according to iTAK
8	*Niben101Scf03493g00018*	2	ATGAACTCCGAGTAGCCACGAGG; GCTATAACCAAACCAGTTCCCGG	
	Niben101Scf02869g18003			
	Niben101Scf01378g00004	2	ATCGGGACGAAAATTCCTAGTGG; GATAGCAATCCAAGCTCGAGGG	
	Niben101Scf00012g00012			
	Niben101Scf06482g03003	3	GTCAGGACGGAAGTTTCTAGTGG; GTAGTAGGAGAAGGAGGTTTTGG	
	Niben101Scf00635g04002			
	Niben101Scf00229g07003			
	Niben101Scf05476g01001	2	TTAAACCAAGAAGGGTGGCAGGG; CTCCTACAACACTATCAGGACGG	
	Niben101Scf01025g10006			
9	*Niben101Scf09004g00018*	2	GTTTATGAGGTCATGACAAGAGG; GCCCATAGTACCTATAACCCTGG	
	Niben101Scf11684g00005	2	TTAAGCCAGAAAGCTTTCAGGGG; GTATATGACCAAAGGAAGCTTGG	
	Niben101Scf05916g00004			
	Niben101Scf10274g00001	2	TTATGGCCCTGCAAACCTCGAGG; GTATATGAATTTATGCCAAGAGG	
	Niben101Scf02826g01012			
	Niben101Scf19533g00007	2	GTCAAGAAATTGAAGCCGGAAGG; GTGTATGAGTTCATGCCTAAAGG	
	Niben101Scf02279g05002			
	Niben101Scf11389g00006	3	GGTTCTGCTGGGAATCCTAGAGG; GCAACTTCGTCATCCAAACCTGG; GTGTATGAGTTCATGCCTAAAGG	
	Niben101Scf01521g13003			

*iTAK (http://itak.feilab.net/); QUT (http://www.benthgenome.qut.edu.au/). The *RLCK* genes that are not expressed according to the QUT genome browser were not chosen to be targeted by CRISPR/Cas9. Some genes that are not present in the phylogenetic tree (Figure S9B) but were found to be clustered in a specific subfamily by using iTAK, were also selected to be knocked out.

Supplementary Table 2. Nucleotide sequences of the primers used in this study.

Primer code	Primer name	Sequence (5' - 3')
Primers used for introducing point mutations in the SOBIR1 kinase domain (the introduced mutations are indicated with a capital).		
ho113	NbSOBIR1_T512A fw*	gcactcccagatgcccatGcacatgttacgacttc
ho114	NbSOBIR1_T512A rev	gaagtcgtaacatgtgCatgggcatctgggagtgc
ho115	NbSOBIR1_T515A fw	gatgcccatacacatgttGcgacttcaaatgttgcagg
ho116	NbSOBIR1_T515A rev	cctgcaacatttgaagtcgCaacatgtgtatgggcatc
ho117	NbSOBIR1_T516A fw	gcccatacacatgttacgGcttcaaatgttgcaggaac
ho118	NbSOBIR1_T516A rev	gttcctgcaacatttgaagCcgtaacatgtgtatgggc
ho119	NbSOBIR1_S517A fw	gcccatacacatgttacgactGcaaatgttgcagg
ho120	NbSOBIR1_S517A rev	cctgcaacatttgCagtcgtaacatgtgtatgggc
ho121	NbSOBIR1_T522A fw	cttcaaatgttgcaggaGctgtgggatatattgcacc
ho122	NbSOBIR1_T522A rev	ggtgcaatatatcccacagCtcctgcaacatttgaag
ao1	SlSOBIR1_T503A fw	gcagttccagatgctcatGcacatatcaccacttc
ao2	SlSOBIR1_T503A rev	gaagtggtgatatgtgCatgagcatctggaactgc
ao3	SlSOBIR1_T506A fw	gctcatacacatatcGccacttcaaatgttgcag
ao4	SlSOBIR1_T506A rev	ctgcaacatttgaagtggCgatatgtgtatgagc
ao5	SlSOBIR1_T507A fw	caccGcttcaaatgttgcaggaactgttggatttattg
ao6	SlSOBIR1_T507A rev	caataaatccaacagttcctgcaacatttgaagCggtg
ao7	SlSOBIR1_S508A fw	gctcatacacatatcaccactGcaaatgttgcagg
ao8	SlSOBIR1_S508A rev	cctgcaacatttgCagtggtgatatgtgtatgagc
ao9	SlSOBIR1_T513A fw	caaatgttgcaggaGctgttggatttattgc
ao10	SlSOBIR1_T513A rev	gcaataaatccaacagCtcctgcaacatttg
ho166	SlSOB-like_T516A_fw	gctgtcccagatgctcatGcacatattacaacttc
ho167	SlSOB-like_T516A_rev	gaagttgtaatatgtgCatgagcatctgggacagc
ho168	SlSOB-like_T519A_fw	cagatgctcatacacatattGcaacttcaaatgtggc
ho169	SlSOB-like_T519A_rev	gccacatttgaagttgCaatatgtgtatgagcatctg
ho170	SlSOB-like_T520A_fw	gatgctcatacacatattacaGcttcaaatgtggcag
ho171	SlSOB-like_T520A_rev	ctgccacatttgaagCtgtaatatgtgtatgagcatc
ho172	SlSOB-like_S521A_fw	ctcatacacatattacaactGcaaatgtggcaggaac
ho173	SlSOB-like_S521A_rev	gttcctgccacatttgCagttgtaatatgtgtatgag
ho174	SlSOB-like_T526A_fw	cttcaaatgtggcaggaGctataggatacatcgctc
ho175	SlSOB-like_T526A_rev	gagcgatgtatcctatagCtcctgccacatttgaag
ho123	NbSOBIR1_Y355F fw	ggtggatgcggagaagtttTtagagctgagttaccggg
ho124	NbSOBIR1_Y355F rev	cccggtaactcagctctaAaaacttctccgcatccacc
ho125	NbSOBIR1_Y426F fw	ctaggccagactgccattTcttggtatatgaatatatg
ho126	NbSOBIR1_Y426F rev	catatattcatataccaagAaatggcagtctggcctag
ho127	NbSOBIR1_Y429F fw	gactgccattacttggtatTtgaatatatgaaaaatgg
ho128	NbSOBIR1_Y429F rev	ccatttttcatatattcaAataccaagtaatggcagtc
ho129	NbSOBIR1_Y431F fw	gccattacttggtatatgaatTtatgaaaaatgggagc
ho130	NbSOBIR1_Y431F rev	gctcccatttttcataAattcatataccaagtaatggc
ho131	NbSOBIR1_Y469F fw	gatagcttctggacttgagtTtctccatataaaccac
ho132	NbSOBIR1_Y469F rev	gtggtttatatggagaAactcaagtccagaagctatc
ho133	NbSOBIR1_Y525F fw	gcaggaactgtgggatTtattgcaccagaataccatc
ho134	NbSOBIR1_Y525F rev	gatggtattctggtgcaataAatcccacagttcctgc
ho135	NbSOBIR1_Y530F fw	gatatattgcaccagaatTccatcagacactgaag

ho136	NbSOBIR1_Y530F rev	cttcagtgtctgatggAattctggtgcaatatatc
ho137	NbSOBIR1_Y543F fw	cgggtaagtgtgatatatTcagctttggtgtggtg
ho138	NbSOBIR1_Y543F rev	caccacaccaaagctgAatatatcacacttacccg
ao11	SlSOBIR1_Y346F fw	gtggctgtggagaagtttTtagagcagagctac
ao12	SlSOBIR1_Y346F rev	gtagctctgctctaAaaacttctccacagccac
ao13	SlSOBIR1_Y417F fw	gccagactgtcattTcttggtctacgaatacatg
ao14	SlSOBIR1_Y417F rev	catgtattcgtagaccaagAaatgacagtctggc
ao15	SlSOBIR1_Y420F fw	cttggtctTcgaatacatgaaaaatgggagtttacag
ao16	SlSOBIR1_Y420F rev	ctgtaaactcccatttttcatgtattcgAagaccaag
ao17	SlSOBIR1_Y422F fw	cgaatTcatgaaaaatgggagtttacaggatatcc
ao18	SlSOBIR1_Y422F rev	ggatatcctgtaaactcccatttttcatgAattcg
ao19	SlSOBIR1_Y460F fw	gctgctggtctcgagtTtctccatataaaccatac
ao20	SlSOBIR1_Y460F rev	gtatggtttatatggagaAactcgagaccagcagc
ao21	SlSOBIR1_Y521F fw	gcaccagaatTtatcagacactgaagtttacag
ao22	SlSOBIR1_Y521F rev	ctgtaaacttcagtgtctgataaAattctggtgc
ao23	SlSOBIR1_Y522F fw	gcaccagaatattTcagacactgaagtttac
ao24	SlSOBIR1_Y522F rev	gtaaacttcagtgtctgaAaatattctggtgc
ao25	SlSOBIR1_Y534F fw	gataagtgtgatatatTcagctttggtgtggtgc
ao26	SlSOBIR1_Y534F rev	gcaccacaccaaagctgAatatatcacacttatc
ao27	SlSOBIR1_Y588F fw	gcttataggaaatggatTcgacgaacaaatgc
ao28	SlSOBIR1_Y588F rev	gcatttgttcgtcgAatccatttcctataagc
ho142	SlSOB-like_Y359F_fw	cattgggcaaggtggatgtggaaaagtttTtaaagctgc
ho143	SlSOB-like_Y359F_rev	ctttccgtcacttccaggtaatgcagctttaAaaacttttcc
ho144	SlSOB-like_Y430F_fw	gccaagaccagactgccactTcttggtctatgagtacatg
ho145	SlSOB-like_Y430F_rev	catgtactcatagaccaagAagtggcagtctggtcttggc
ho146	SlSOB-like_Y433F_fw	ctgccactacttggtctTtgagtacatgaaaaatgggagc
ho147	SlSOB-like_Y433F_rev	gctcccatttttcatgtactcaAagaccaagtagtggcag
ho148	SlSOB-like_Y435F_fw	gccactacttggtctatgagtTcatgaaaaatgggagc
ho149	SlSOB-like_Y435F_rev	gctcccatttttcatgAactcatagaccaagtagtggc
ho150	SlSOB-like_Y473F_fw	gctgctggactcgagtTtctccatataaatcatactcagcg
ho151	SlSOB-like_Y473F_rev	cgctgagtatgatttatatggagaAactcgagtccagcagc
ho152	SlSOB-like_Y529F_fw	ggcaggaactataggatTcatcgctccagaatatcatcag
ho153	SlSOB-like_Y529F_rev	ctgatgatattctggagcgatgAatcctatagttcctgcc
ho154	SlSOB-like_Y534F_fw	ggatacatcgctccagaatTtcatcagacactgaagttcactg
ho155	SlSOB-like_Y534F_rev	cagtgaacttcagtgtctgatgaAattctggagcgatgtatcc
ho156	SlSOB-like_Y547F_fw	ctgataagtgtgatatatTcagctttggggtgctg
ho157	SlSOB-like_Y547F_rev	cagcaccccaaagctgAatatatcacacttatcag
ho447	1004/5004_K110A_f2	ggctcagcctgtggctgtaGCgcttttggatttag
ho448	1004/5004_K110A_r2	ctaaatccaaaagcGCtacagccacaggctgagcc
ho451	13012_K127A-f2	gctgttGCaaaatcaaatcctgatagtgaacaaggtctcaagg
ho452	13012_K127A-r2	gatttgattttGCaacagcaactggaattccaactccaactttag
ho449	1025_K119A-f2	gtaattgctgttGCgaaattgaattccgaaagcttacaggggttg
ho450	1025_K119A-r2	caatttcCGaacagcaattacagtgccgctgccactcc
ho453	00012_K109A-f2	gtgtgatcattgctgtgGCacggctgaatcaagaag
ho454	00012_K109A-r2	cttcttgattcagccgtGCcacagcaatgatcacac
ho472	6482_K114A-f2	gctgtgGCgaagttaaatcaagaaggtttgcaggggcac
ho473	6482_K114A-r2	gatttaacttcGCcacagctatcacgattccagctccagg
Primers for genotyping the *rlck-vii-6* knock-out plants.		
ho228	747_geno_1-F_JS2419	ccaaaaccaaaggcaaaggag

ho229	747_geno_1-R1004_JS2420	gattagccaggtagatagtgg
ho230	747_geno_1-R5004_JS2421	gactgaagttccaaaagcc
ho231	747_geno_2-F_JS2422	gtgatgaagattgcatggg
ho232	747_geno_2-R2008_JS2423	gtttacgagttattgcacgat
ho233	747_geno_2-R3011_JS2424	caccaactcccaaaagcc
ho234	747_geno_3-F3002_JS2425	gtggtcattcaaggctagc
ho235	747_geno_3-R3002_JS2426	ttttcttaaactccgtgccg
ho236	747_geno_4-F13015_JS2427	tcatcatcatagtaggctagcc
ho237	747_geno_4-R13015_JS2428	tagtccttttgcagcaccta
ho238	747_geno_5-F_JS2429	cgtaggttgtcattctcgga
ho239	747_geno_5-R1006_JS2430	tgtaacttcttggggtacgt
ho240	747_geno_5-R0005_JS2431	tgcgtaccatcaaaacaacc
ho241	747_R1-13015_JS2486	tgttttctcttacacacccc
ho242	747_F2-13015_JS2487	tgaatacatggcaaggggaa
Primers for genotyping the *rlck-vii-7* knock-out plants.		
LHo07	734_00009F_JS2556	ccagttcttctggtctacg
LHo08	734_03007F_JS2555	cctgttcttctgatctatg
LHo09	734_03007R+00009R_JS2557	caactaagcagaccttacc
LHo10	734_01025F_JS2558	tccattgttcagacacagtg
LHo11	734_06004F_JS2559	cattgctcagagatagtgag
LHo12	734_06004R+01025R_JS2560	gatgctcagaaagaagagg
LHo13	734_02015F_JS2551	acaaaaggacccaaattgct
LHo14	734_02015R_JS2552	tggagtaagcgtcttctcat
LHo15	734_03005F_JS2553	ctacgtacgcctcttacaga
LHo16	734_03005R_JS2554	cggtgattgcttgtatcgat
LHo17	734_13012F_JS2561	tagcaacagcacaacttgtt
LHo18	734_13012R_JS2562	tccttgagaccttgttcact
Primers for genotyping the *rlck-vii-8* knock-out plants.		
Po05	Scf00012	aaattagaacacggacaat
Po01	Scf00229_fw	gctgcaaagatagtagcaattgg
Po02	Scf00229_rv	gaagcacgcagataggcg
Po03	Scf00635_fw	gcaattggcttttgtatgtgc
Po04	Scf00635_rv	gcgacctaataatagttcaaaattgc
Po36	Scf1025_fw	gactagtaattcgaacagc
Po37	Scf1025_rv	tccctaatctgtatatgggg
Po09	Scf01378	aaattaggacacgggcaac
Po08	Scf02819	tttcggttgaacccgtgga
Po10	Scf03493	tttcggttgaactcatagc
Po11	Scf05476_fw	gtaattggtttctttggatgtgtatc
Po14	Scf05476_rv	gcgaatcaagtcgcctattcatca
Po15	Scf6482_fw	ggtgtcatgagaagtgcagattgc
Po16	Scf6482_fw	ccaattttactacgaagtaccatgcg
Po06	Scf06482_fw	gcaaagatagtagtaattggcttttgtatgtgc
Po07	Scf06482_rv	gcaaatgaatgtaattttgaactagtattacgtgc
Po12	Scf0012-1378_fw	atgttgctatctctggttt
Po13	Scf02869-3493_rv	gcctgtgttcatcttctaag
Primers used for generating *E. coli* expression constructs of the SOBIR1 kinase domain (sequences that are identical to the expression vectors are underlined).		

ho201	pET-GST_fw	aagcttgcggccgcactcgag
ho202	pET-GST_rev	caggggcccctggaacagaacttc
ho203	NbSOBIR1_KD+90bp_fw	gttctgttccaggggcccctgcgaaagggaaagactgatggaac
ho204	NbSOBIR1_KD+90bp_rev	ctcgagtgcggccgcaagcttctaatgcttgatctgagttaacatac
ho215	SlSOBIR1_KD+90bp_fw	gttctgttccaggggcccctgagaagagggaataacgattcaag
ho216	SlSOBIR1_KD+90bp_rev	ctcgagtgcggccgcaagcttctaatgcttgatctgagttaacatgc
ho217	SlSOBIR1-like_KD+90bp_fw	gttctgttccaggggcccctgagagggatcagaaatgatccagg
ho218	SlSOBIR1-like_KD+90bp_rev	ctcgagtgcggccgcaagcttttaatgcttgatctgcatcaacatgc
ho219	pET-15b_fw	cggatcctcgagcatatggctg
ho220	pET-15b_rev	gctgctaacaaagcccgaaaggaag
ho221	NbSO_KD+90bp_fw	gccatatgctcgaggatccgcgaaagggaaagactgatggaac
ho222	NbSO_KD+90bp_rev	ctttcgggctttgttagcagcctaatgcttgatctgagttaacatac
ho223	SlSO_KD+90bp_fw	gccatatgctcgaggatccgagaagagggaataacgattcaag
ho224	SlSO_KD+90bp_rev	ctttcgggctttgttagcagcctaatgcttgatctgagttaacatgc
ho225	SlSO-like_KD+90bp_fw	gccatatgctcgaggatccgagagggatcagaaatgatccagg
ho226	SlSO-like_KD+90bp_rev	ctttcgggctttgttagcagcttaatgcttgatctgcatcaacatgc
ho282	NbSERK3b_kd_fw	gccatatgctcgaggatccgggacaactcaagaggttttccttg
ho283	NbSERK3b_kd_rev	ctttcgggctttgttagcagctcatcttggccccgacaattcatc
ho286	SlSERK3a_kd_fw	gccatatgctcgaggatccgggacaactcaaaaggttttccttgc
ho287	SlSERK3a_kd_rev	ctttcgggctttgttagcagctcatcttggccctgacaactcatc
ho326	1004-5004GST_fw	gttctgttccaggggcccctgatgaagattacatgggaatctctagttcc
ho327	1004GST_rv	ctcgagtgcggccgcaagcttagctctatggaaatcattgtgcaatg
ho333	5004GST_rv	ctcgagtgcggccgcaagcttagctctatggaaattattgcgcaatg
ho439	13012_GST-fw	gttctgttccaggggcccctgatgggaaattgctggcctaaatc
ho440	13012_GST-rev	ctcgagtgcggccgcaagcttgtagcttcgattgatggcagcatg
ho437	1025_GST-fw	gttctgttccaggggcccctgatgggaatttgtttcagtaatgcgtc
ho438	1025_GST-rev	ctcgagtgcggccgcaagcttgtatgctcttcttgggagtggatag
ho441	00012_GST-fw	gttctgttccaggggcccctgatggggatttgtttgagtgctag
ho442	00012_GST-rev	ctcgagtgcggccgcaagcttttagcataaaagggagaagc
ho464	6482_GST_fw	gttctgttccaggggcccctgatggggatttgtctgagcaacc
ho465	6482_GST_rev	ctcgagtgcggccgcaagcttctgcccggggagtctcttc
Primers used for generating the split-luciferase binary constructs.		
JRO03	KpnI-Solyc07g041940.2-fw	cggggtaccatgaagattggatgggaatc
JRO04	SalI-Solyc07g041940.2-rev	acgcgtcgacggatctcttgaaaccattttg
JRO05	KpnI-Solyc04g082500.2-fw	cggggtaccatgggaaattgttggcc
JRO06	SalI-Solyc04g082500.2-rev	acgcgtcgacatagctacggtttttggagc
JRO07	KpnI-Solyc11g062400.1-fw	cggggtaccatggggatttgctttgg
JRO08	SalI-Solyc11g062400.1-rev	acgcgtcgactcttgaatgtgtcacatatctctt
JRO09	KpnI-Solyc06g062920.2-fw	cggggtaccatggctacttgcggaattg
JRO10	SalI-Solyc06g062920.2-rev	acgcgtcgacataagaatttagtttgtttgttctttc
JRO11	KpnI-Solyc05g025820.2-fw	cggggtaccatgaagattacatgggaatctctag
JRO12	SalI-Solyc05g025820.2-rev	acgcgtcgacagctctatggaaatcattgtgc
JRO13	KpnI-Solyc01g088690.2-fw	cggggtaccatgaagtgtttttttacttcaag
JRO14	XhoI-Solyc01g088690.2-rev	ccgctcgagtgtagttttacccttctgcatg
JRO15	KpnI-Solyc05g007050.2-fw	cggggtaccatgggattaggtggtgatg
JRO16	SalI-Solyc05g007050.2-rev	acgcgtcgacagttttgccgtttggttt
JRO17	KpnI-Solyc08g077560.2-fw	cggggtaccatgctgaagtgtttttatatattca
JRO27	SalI-Solyc08g077560.2-rev	acgcgtcgacagcctgagctaagg
JRO21	KpnI-Solyc01g112220.2-fw	cggggtaccatgggagtttgtttcagttctaa

JRO22	SalI-Solyc01g112220.2-rev	acgcgtcgactgatgctctttttgggagtg
JRO23	KpnI-Solyc06g005500.2-fw	cggggtaccatggggatatgtttgagtgc
JRO24	SalI-Solyc06g005500.2-rev	acgcgtcgactttagcgtaaaggggagaag
Lo94	SlSOBIR1-Kpn1-fw	cggcgcggtaccatgacttcgaatatccactttttcttttatacg
Lo95	SlSOBIR1-Sal1-NoStop-rev	gccgccgtcgacatgcttgatctgagttaacatgc
Lo153	GUS-Kpn1-fw	cggcggggtaccatgttacgtcctgtagaaacc
Lo154	GUS-Sal1-rev	gaagccgtcgacttgtttgcctccctgctgcggtttttc
Primers used for generating the TbID binary constructs.		
JO4	NbSOBIR1-fw	ttcttgaaggtcatgtgcc
JO6	YFP-rev	ctcaggtagtggttgtcgg
JO8	TurboID-rev	gcagatacaggtcattggg
JO9	GUS-fw	ggttatctctatgaactgtgcg
Primers used for generating the complementation construct of Niben101Scf00012g00012.1. Nucleotides in lower case letters align with the Gateway sequence.		
Po17	Niben101Scf00012g00012.1 fw	caccATGGGGATTTGTTTGAGTGCTAG
Po18	Niben101Scf00012g00012.1 fw	TTTAGCATAAAAGGGAGAAGC

* fw, forward; rev, reverse.
