# Supplementary material for: Receptor-like cytoplasmic kinases of different subfamilies differentially regulate SOBIR1/BAK1-mediated immune responses in Nicotiana benthamiana
Source: Nat Commun. 2024 May 21;15:4339. doi: 10.1038/s41467-024-48313-1 (PMC11109355; doi:10.1038/s41467-024-48313-1)
Supplement: Supplementary file 2 — Reporting Summary [file 41467_2024_48313_MOESM2_ESM.pdf]

nature portfolio

Corresponding author(s): Wen R.H. Huang
Matthieu H.A.J. Joosten

Last updated by author(s): Apr 20, 2024

Reporting Summary

Nature Portfolio wishes to improve the reproducibility of the work that we publish. This form provides structure for consistency and transparency in reporting. For further information on Nature Portfolio policies, see our Editorial Policies and the Editorial Policy Checklist.

Statistics

For all statistical analyses, confirm that the following items are present in the figure legend, table legend, main text, or Methods section.

n/a	Confirmed	
☐	☒	The exact sample size (*n*) for each experimental group/condition, given as a discrete number and unit of measurement
☐	☒	A statement on whether measurements were taken from distinct samples or whether the same sample was measured repeatedly
☐	☒	The statistical test(s) used AND whether they are one- or two-sided<br>*Only common tests should be described solely by name; describe more complex techniques in the Methods section.*
☒	☐	A description of all covariates tested
☐	☒	A description of any assumptions or corrections, such as tests of normality and adjustment for multiple comparisons
☐	☒	A full description of the statistical parameters including central tendency (e.g. means) or other basic estimates (e.g. regression coefficient) AND variation (e.g. standard deviation) or associated estimates of uncertainty (e.g. confidence intervals)
☐	☒	For null hypothesis testing, the test statistic (e.g. *F*, *t*, *r*) with confidence intervals, effect sizes, degrees of freedom and *P* value noted<br>*Give P values as exact values whenever suitable.*
☒	☐	For Bayesian analysis, information on the choice of priors and Markov chain Monte Carlo settings
☒	☐	For hierarchical and complex designs, identification of the appropriate level for tests and full reporting of outcomes
☒	☐	Estimates of effect sizes (e.g. Cohen's *d*, Pearson's *r*), indicating how they were calculated

Our web collection on statistics for biologists contains articles on many of the points above.

Software and code

Policy information about availability of computer code

Data collection	Protein sequences of the SOBIR1 homologue from various plant species were obtained from the UniProt database (https://www.uniprot.org/).<br>Protein sequences of RLKs were retrieved from TAIR (https://www.arabidopsis.org/index.jsp) and the Sol Genomics Network (https://solgenomics.net/).<br>The predicted proteomes of Nicotiana benthamiana, tomato and Arabidopsis were obtained from www.solgenomics.net and www.arabidopsis.org. Necrotic areas of Phytophthora palmivora inoculation were determined by using Chemidoc Imaging System (Bio-Rad).
Data analysis	Image Lab: 6.0.1<br>GraphPad Prism: 9.3.1<br>Jalview: 2.11.2.7<br>HMMER: v3.1b2<br>MAFFT: v7.271<br>ClipKIT: v1.3.0<br>IQ-Tree: v2.2.0

For manuscripts utilizing custom algorithms or software that are central to the research but not yet described in published literature, software must be made available to editors and reviewers. We strongly encourage code deposition in a community repository (e.g. GitHub). See the Nature Portfolio guidelines for submitting code & software for further information.

Data

Policy information about availability of data

All manuscripts must include a data availability statement. This statement should provide the following information, where applicable:

- Accession codes, unique identifiers, or web links for publicly available datasets
- A description of any restrictions on data availability
- For clinical datasets or third party data, please ensure that the statement adheres to our policy

All data are available within this Article and its Supplementary Information. Source Data (original gel blots and numerical data) are provided in this article. The data availability statement in the manuscript is the following: "The authors declare that all data supporting the findings of this study are available within the manuscript and the Supplementary Files or are available from the corresponding authors upon request. Source data are provided with this paper.".

Research involving human participants, their data, or biological material

Policy information about studies with human participants or human data. See also policy information about sex, gender (identity/presentation), and sexual orientation and race, ethnicity and racism.

Reporting on sex and gender	There are no human participants or human data in this study.
Reporting on race, ethnicity, or other socially relevant groupings	There are no human participants or human data in this study.
Population characteristics	There are no human participants or human data in this study.
Recruitment	There are no human participants or human data in this study.
Ethics oversight	There are no human participants or human data in this study.

Note that full information on the approval of the study protocol must also be provided in the manuscript.

Field-specific reporting

Please select the one below that is the best fit for your research. If you are not sure, read the appropriate sections before making your selection.

☒ Life sciences ☐ Behavioural & social sciences ☐ Ecological, evolutionary & environmental sciences

For a reference copy of the document with all sections, see nature.com/documents/nr-reporting-summary-flat.pdf

Life sciences study design

All studies must disclose on these points even when the disclosure is negative.

Sample size	Sample size was determined based on experimental trials and with consideration of previous publications on similar experiments to allow for confident statistical analyses. No statistical methods were used to predetermine sample size.<br>Previous publications considered to determine sample size:<br>HR assay in Nicotiana benthamiana (doi:10.1093/plphys/kiaa047; DOI: 10.1111/mpp.13027)<br>ROS assay (doi:10.1093/plphys/kiaa047)<br>MAPK activation assay (https://doi.org/10.1094/MPMI-08-17-0203-FI)<br>In vitro phosphorylation assay (http://www.plantmethods.com/content/9/1/22)<br>Phytophthora palmivora inoculation assay (https://doi.org/10.1128/mBio.01516-19)
Data exclusions	No data were excluded from the analyses provided.
Replication	Reproducibility of data was tested by multiple repetitions of the experiments described. Each experiments were repeated at least three times independently. Results were reproducible with the same trend. At least three technical replicates were included in the individual biological replicate experiments.
Randomization	Plants of different genotypes were grown side by side in the same growth chamber to avoid the environmental effects. Leaf samples of similar age were collected randomly for all experiments.
Blinding	The investigator were not blinded to group allocation during experiment performing and outcome assessment. The nature of the experiments conducted in this study requires that the investigators know precisely what plants have received what treatment. Experiments were repeated by different authors, whenever possible.

Reporting for specific materials, systems and methods

We require information from authors about some types of materials, experimental systems and methods used in many studies. Here, indicate whether each material, system or method listed is relevant to your study. If you are not sure if a list item applies to your research, read the appropriate section before selecting a response.

Materials & experimental systems

n/a	Involved in the study	
☐	☒	Antibodies
☒	☐	Eukaryotic cell lines
☒	☐	Palaeontology and archaeology
☒	☐	Animals and other organisms
☒	☐	Clinical data
☒	☐	Dual use research of concern
☐	☒	Plants

Methods

n/a	Involved in the study	
☒	☐	ChIP-seq
☒	☐	Flow cytometry
☒	☐	MRI-based neuroimaging

Antibodies

Antibodies used

Commercial antibodies used:
anti-p42/p44-erk (Cell signaling, Cat. # 9101, 1:2000)
anti-Rabbit IgG (Sigma Aldrich, Cat. #A6154. 1:10000)
anti-SOBIR1 (Agrisera, Cat. #AS16 3204, 1:5000)
anti-GFP-HRP (Miltenyi Biotec, Cat. #130-091-833, 1:5000)
anti-Cluc (1 µg/mL) (Sigma, L2164)
anti-mouse-HRP (1:10,000) (GE Healthcare)

Validation

Validation information and experiments can be obtained from the following websites and publications:
anti-p42/p44-erk (https://www.cellsignal.com/products/primary-antibodies/phospho-p44-42-mapk-erk1-2-thr202-tyr204-antibody/9101) (DOI : 10.1111/mpp.12767).
anti-SOBIR1 (https://www.agrisera.com/en/artiklar/sobir1-suppressor-of-bir1.html) (https://www.arabidopsis.org/servlets/TairObject?id=32371&type=locus).
anti-GFP-HRP (https://www.miltenyibiotec.com/NL-en/products/gfp-antibody-gg4-2c2-12-10.html) (DOI: 10.1111/mpp.12789).
anti-Cluc and anti-mouse-HRP (https://www.sigmaaldrich.com/GB/en/product/sigma/l2164)

Dual use research of concern

Policy information about dual use research of concern

Hazards

Could the accidental, deliberate or reckless misuse of agents or technologies generated in the work, or the application of information presented in the manuscript, pose a threat to:

No	Yes	
☒	☐	Public health
☒	☐	National security
☒	☐	Crops and/or livestock
☒	☐	Ecosystems
☒	☐	Any other significant area

Experiments of concern

Does the work involve any of these experiments of concern:

No	Yes	
☒	☐	Demonstrate how to render a vaccine ineffective
☒	☐	Confer resistance to therapeutically useful antibiotics or antiviral agents
☒	☐	Enhance the virulence of a pathogen or render a nonpathogen virulent
☒	☐	Increase transmissibility of a pathogen
☒	☐	Alter the host range of a pathogen
☒	☐	Enable evasion of diagnostic/detection modalities
☒	☐	Enable the weaponization of a biological agent or toxin
☒	☐	Any other potentially harmful combination of experiments and agents

Plants

Seed stocks	N. benthamiana wild type; N. benthamiana:Cf-4 (DOI: 10.1094 /MPMI -19-0567); N. benthamiana:Cf-4 sobir1 knock-out lines (doi:10.1093/plphys/kiaa047).
Novel plant genotypes	All the rlck-vii knock-out plants used in this study were generated by the authors. The method used to knock out these genes has been described previously (doi: 10.1111/tpj.15197). The selection of genes to knock out, genotyping and phenotyping of knock-out plants were described in this article.
Authentication	The genotypes of rlck-vii-6, rlck-vii-7 and rlck-vii-8 knock-out plants were determined by amplifying the sgRNA-targeted gene regions and Sanger sequencing of the obtained PCR fragments. Multiple independent homozygous knock-out lines were selected with same genes being knocked out. Results are shown in the Supplemental Information.
